# GraphscoreDTA: optimized graph neural network for protein–ligand binding affinity prediction

**DOI:** 10.1093/bioinformatics/btad340

**Published:** 2023-05-24

**Authors:** Kaili Wang, Renyi Zhou, Jing Tang, Min Li

**Affiliations:** School of Computer Science and Engineering, Central South University, Changsha 410083, China; School of Computer Science and Engineering, Central South University, Changsha 410083, China; Research Program in Systems Oncology, University of Helsinki, 00014 Helsinki, Finland; Department of Biochemistry and Developmental Biology, University of Helsinki, 00014 Helsinki, Finland; School of Computer Science and Engineering, Central South University, Changsha 410083, China; Hunan Provincial Engineering Research Center of Intelligent Computing in Biology and Medicine, Changsha 410083, China

## Abstract

**Motivation:**

Computational approaches for identifying the protein–ligand binding affinity can greatly facilitate drug discovery and development. At present, many deep learning-based models are proposed to predict the protein–ligand binding affinity and achieve significant performance improvement. However, protein–ligand binding affinity prediction still has fundamental challenges. One challenge is that the mutual information between proteins and ligands is hard to capture. Another challenge is how to find and highlight the important atoms of the ligands and residues of the proteins.

**Results:**

To solve these limitations, we develop a novel graph neural network strategy with the Vina distance optimization terms (GraphscoreDTA) for predicting protein–ligand binding affinity, which takes the combination of graph neural network, bitransport information mechanism and physics-based distance terms into account for the first time. Unlike other methods, GraphscoreDTA can not only effectively capture the protein–ligand pairs’ mutual information but also highlight the important atoms of the ligands and residues of the proteins. The results show that GraphscoreDTA significantly outperforms existing methods on multiple test sets. Furthermore, the tests of drug–target selectivity on the cyclin-dependent kinase and the homologous protein families demonstrate that GraphscoreDTA is a reliable tool for protein–ligand binding affinity prediction.

**Availability and implementation:**

The resource codes are available at https://github.com/CSUBioGroup/GraphscoreDTA.

## 1 Introduction

Identification of high binding affinity values for candidate small molecules plays an important role in drug discovery and drug design (Li *et al.* 2020, Zheng *et al.* 2020). The protein–ligand pair’s affinity refers to the interaction binding strength, the larger value indicating a higher binding affinity, which is usually expressed by experimentally determined inhibition constant $K_{i}$, dissociation constant $K_{d}$, and half-maximal inhibitory concentration ${IC}_{50}$. At present, various experimental methods including isothermal titration calorimetry (Velazquez-Campoy and Freire 2006) and surface plasmon resonance (Maynard *et al.* 2009) can be used for protein–ligand binding affinity measurement. However, these conventional experimental methods are costly and time-consuming. Therefore, it is urgent to develop efficient computational methods capable of making accurate protein–ligand binding affinity prediction (Cao and Li 2014, Wang *et al.* 2021, 2022, Zhu *et al.* 2023). The methods for protein–ligand binding affinity prediction can be easily classified into two classes according to the information employed (Cang and Wei 2018, Jiménez *et al.* 2018, Jiang *et al.* 2020, Wee and Xia 2021). The first class is the sequence-based methods that use the available sequences as the input. For example, Öztürk *et al.* (2018) proposed a 1D convolutional neural networks (CNNs)-based model to learn the features of protein and small molecule. Wang et al. developed a deep learning model with a combination of traditional and dilated convolutional layers. The captured short-range and long-range interactions from the pocket, protein and ligand sequence contextual can contribute to the binding affinity determination (Wang *et al.* 2021). The second class is structure-based methods that predict the binding affinity using the known structures. For example, Stepniewska-Dziubinska *et al.* (2018) proposed a 3D CNN to learn the interaction information from molecule complex. Seo *et al.* (2021) developed a model consisting of both convolutional layer and attention layer to capture the descriptor information. Moreover, Jones *et al.* (2021) proposed a fusion model to concatenate feature representations from two independent trained models [3D-CNN and graph neural network (GNN)]. The sequence-based approaches do not require protein–ligand structures, however, since the actual protein–ligand interaction occurs in 3D, using 3D structure shall overcome information lost for predicting the protein–ligand binding affinity. Meanwhile, the existing structure-based methods typically ignore either global information or local information, and most of these models focus only on individual molecule of a protein–ligand pair, while ignoring their mutual information.

In this study, we employed a novel GNN strategy by adding the Vina distance optimization terms (GraphscoreDTA). Considering the interactions between small molecules and proteins, our model also includes a mutual information mechanism to learn and exchange the representations of proteins and small molecules. Unlike existing graph neural networks, GraphscoreDTA mainly has the following novelties: (i) bitransport information mechanism is constructed to bridge the gap between protein and ligand feature extraction; (ii) skip connections are employed in the GNNs to avoid gradient vanish problem; (iii) multihead attention mechanism is designed to weigh the contributions from individual atoms of a ligand and individual residues of a protein, respectively; (iv) gated recurrent units (GRUs) are introduced to dominate the information proportion between the graph general nodes and super nodes; and (v) Vina distance terms are used to optimize the model prediction. To further evaluate the generalization ability of GraphscoreDTA, two variants of the model were trained based on compound and protein similarity splitting strategy, respectively. The results showed that the models have better performances on the test sets compared with the state-of-the-art methods. In particular, we showed that (i) employing 8 Å as the cutoff of protein contact map may help capture both long-range interactions and short-range interactions; (ii) our model can identify important residues and atoms for proteins and ligands, which may reveal the recognition mechanisms among protein–ligand pairs; and (iii) the modeling structures are predictive even when the number of experimentally solved protein structures is insufficient. Furthermore, we showed the potential of GraphscoreDTA for predicting novel inhibitors for disease-related targets.

## 2 Materials and methods

### 2.1 Datasets

**Figure 1.**
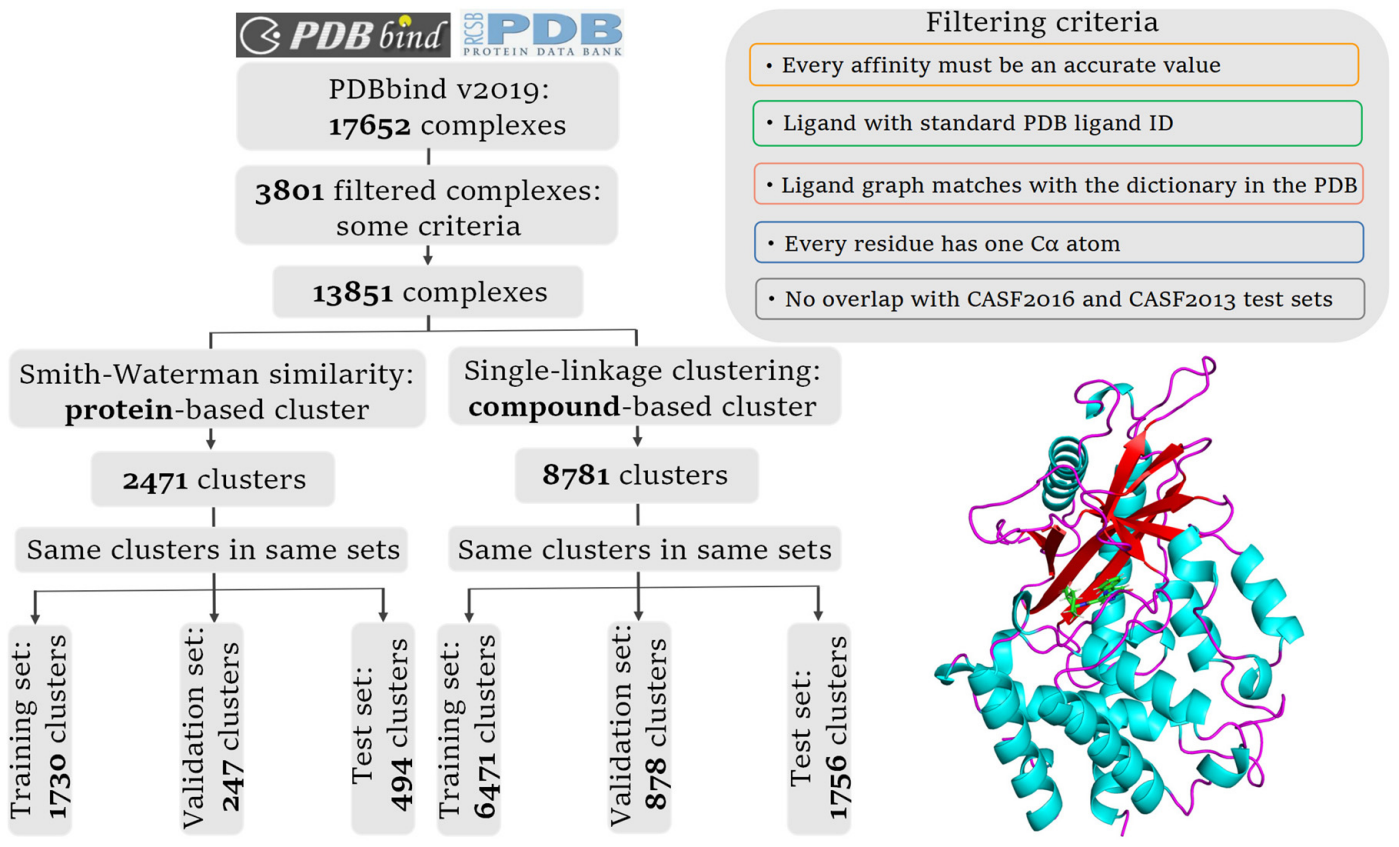
Pipeline to construct the datasets

In this study, PDBbind (version 2019) database (Wang *et al.* 2004) was used to benchmark protein–ligand binding affinity prediction. This database is a comprehensive collection of 17,652 protein–ligand pairs deposited in the PDB (Burley *et al.* 2021), for which the binding affinities were obtained from multiple readouts such as *K*_i_, *K*_d_, and IC_50_ (Fig. 1). The measured values were further transformed into log space in the unit of *M* and expressed as pKa (−log*K*_d_, −log*K*_i_, or −logIC_50_). In addition, the other two high quality test sets, namely, CASF2016 benchmark (Su *et al.* 2019) comprising 285 protein–ligand complexes and CASF2013 benchmark (Li *et al.* 2018) including 195 protein–ligand complexes, were further used to evaluate the model performance. The two test sets are classical benchmarks including diversiform structures and binding affinity data for various docking methods evaluation. Then, the protein–ligand pairs were carefully checked and filtered using the following criteria including: (i) every measurement should be a specifical value, rather than an approximation or a range; (ii) only ligands with standard PDB ID will be remained, while ligands with names like “4-mer” will be discarded; (iii) ligand graphs should be matched between the PDBbind database and the chemical component dictionaries in the PDB databank; (iv) only the proteins with one Cα atom in each residue will be remained; and (v) the protein–ligand pairs in PDBbind database do not overlap with the complexes in CASF2016 and CASF2013 benchmarks. As a result, 13,851 protein–ligand complexes in PDBbind database, 279 complexes in CASF2016 benchmark, and 182 complexes in CASF2013 benchmark were selected.

We split the 13,851 data into disjoint training, validation and test sets to prevent data leakage. Clustering-based methods were employed to ensure that similar proteins or compounds in the same cluster were assigned into the same set. Specifically, the Smith–Waterman similarity score (Zhao *et al.* 2013) and single-linkage clustering distance (Gower and Ross 1969) were calculated to distinguish dissimilar proteins and compounds, respectively. The clustering threshold was defined as 0.3. Next, these protein–ligand pairs were divided, where the ratios of clusters in the training, validation and test sets were ∼7:1:2 (Supplementary Table S1).

### 2.2 Feature representation

In our architecture, the protein 3D structures were transformed into graphs, in which the amino acids in the given protein were defined as nodes and two nonconsecutive amino acids were connected by an edge. For compound graphs, nodes denote atoms and edges represent chemical bonds. Similarly, the interaction-based subgraphs were constructed to represent pocket–ligand interaction information. More details are described in Supplementary Section S1.

### 2.3 Model construction

**Figure 2.**
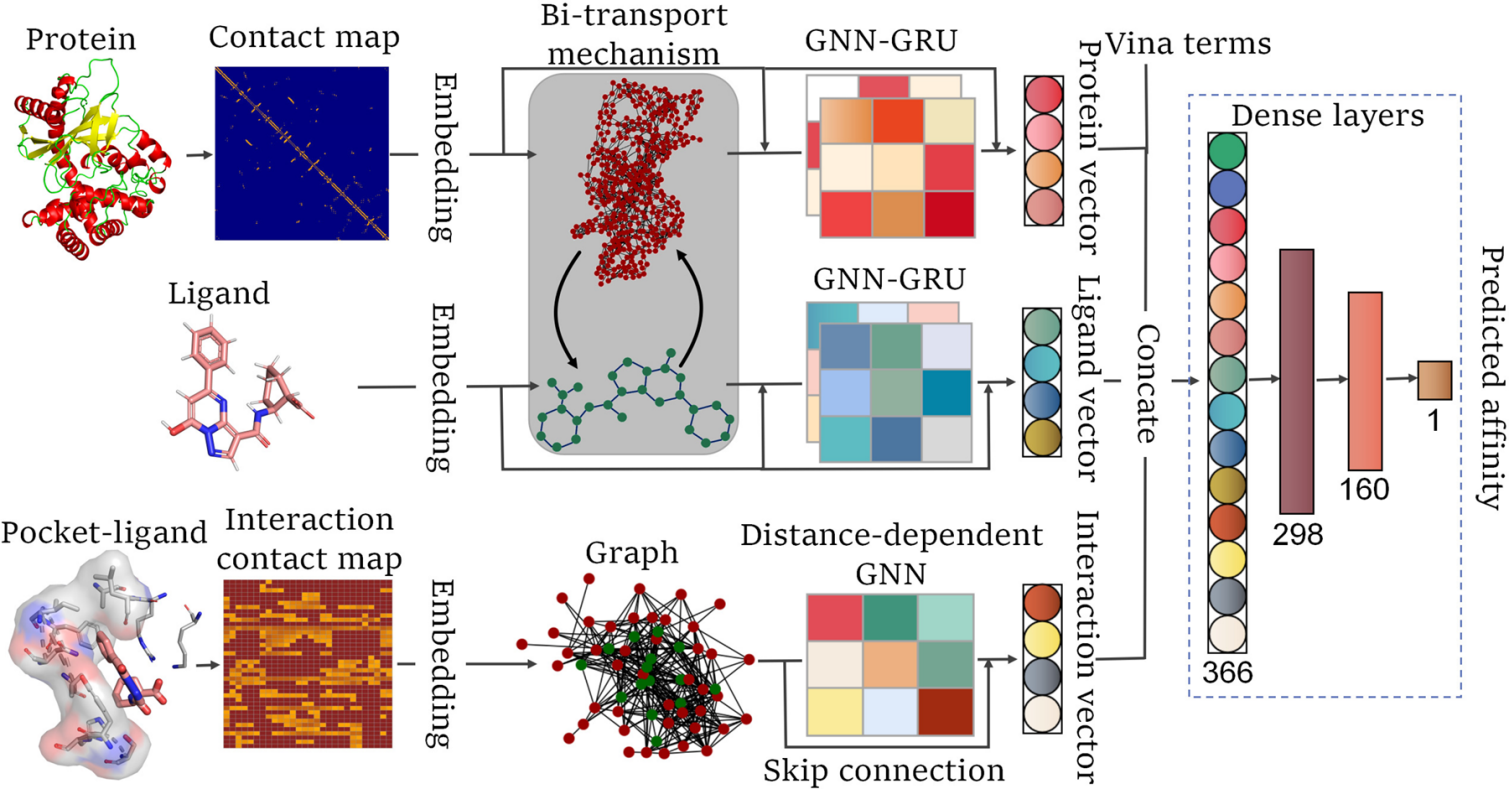
Architecture of GraphscoreDTA. Three main GNNs blocks were constructed to learn features from protein structures, ligand structures, and pocket–ligand interaction structures, respectively. In the protein and ligand blocks, bitransport information mechanism was designed to bridge the gap between the protein and ligand. Then GNNs were constructed to learn the protein and ligand structure features, respectively. Multihead attention mechanism, GRUs, and skip connection were introduced in the GNNs. In the pocket–ligand interaction block, the distance-dependent GNNs were constructed to learn the pocket–ligand structure features. Finally, the learned features from three blocks and the Vina distance optimization terms were connected and fed into three dense layers to predict the binding affinity

In this work, we developed a graph neural network-based architecture with Vina distance optimization terms to predict the binding affinity. As shown in Fig. 2, the embedding layers were first employed to transform the node/edge vector into the fixed dimension representation. Next, three main GNN blocks were constructed to learn features from protein structures, ligand graphs and pocket–ligand interaction structures, respectively. More specifically, the details of the constructed GNN are listed as follows. First, bitransport information mechanism was introduced to perform information transfer between proteins and ligands. Then, the amino acid/atom features were processed by two iterations of protein/compound GNN to get the updated amino acid/atom features. In the second iteration, one skip connection was added between embedding vector and input of the GNN, then another skip connection was added between embedding vector and output of the GNN. In the interaction graph, the amino acid and atom features were processed by one interaction of GNN to get the updated features, after which one skip connection was employed between embedding vector and output of the GNN. Next, multihead attention mechanism was introduced into ligand and protein GNNs to weigh the contributions from individual atoms of ligand and individual residues of protein, respectively. Moreover, GRUs were used to dominate the information proportion between general node and super node of the ligand and protein graphs, respectively. Furthermore, Vina distance terms which characterize intermolecular interactions and ligand contributions were employed to optimize the model predictive ability. We used the scoring function terms of AutoDock Vina, which is a popular program for molecular docking and virtual screening. Here, the intermolecular Vina terms include three steric interactions (gauss1, gauss2, and repulsion), hydrophobic bond term, and hydrogen bond term. The ligand flexible term is the number of active rotatable bonds between heavy atoms of the ligand. More details about the optimization terms are shown in Supplementary Section S2 (Trott and Olson 2010, Hongjian *et al.* 2014). Finally, the learned representations from three graph neural network blocks and the Vina distance optimization terms were connected and further fed into three dense layers, where 390 and 320 nodes were included in the first two dense layers, each followed by the activation function PReLU. The third layer with 160 nodes was followed by the output. To evaluate model performance, the validation set was used to tune and identify the model parameters. The training process was repeated 100 times and the model with the best performance on validation set was selected. The hyperparameters are listed in Supplementary Table S2.

#### 2.3.1 Bitransport information mechanism

**Figure 3.**
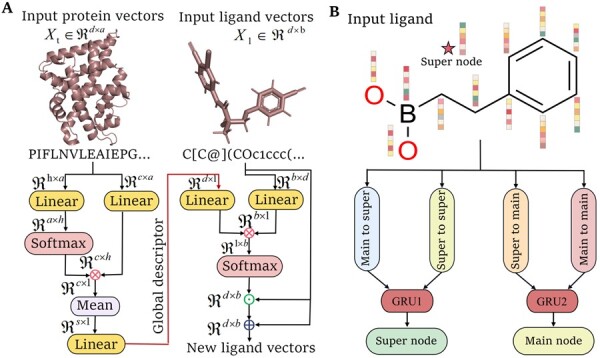
Architectures of bitransport information mechanism (A) and graph neural network for the ligands (B)

Here, the bitransport information mechanism (Fig. 3A) was employed to bridge the gap between protein and ligand features extraction. Bitransport information mechanism was mainly achieved by the multihead attention and position-aware attention. For ligand features update, the multihead attention was first used to convert the protein feature vector $X_{p}\in R^{n\times c}$ into a fixed global descriptor representation $G_{p}\in R^{1\times c}$. In this work, we employed h=8 parallel attention heads. The protein global descriptor representation is defined as where $w_{g}$ is a learnable parameter, *c* is the feature’s dimension. $g_{i,i=1,2,\ldots ,h}$ is defined as where *T* can be obtained by transforming the $X_{p}$ as where $w_{p}$ is a learnable parameter. Furthermore, the multihead attention can be defined as each attention weight ${e_{i}}^{j}$ in position *j* is computed by where *s* is the feature’s dimension, and *n* is the number of amino acids of protein.


(1) $$G_{p}=w_{g}\left(\frac{1}{h}\sum_{i}g_{i}\right)\in R^{1\times c},\;$$

(2) $$g_{i}=Te_{i}\in R^{s\times 1},\;$$

(3) $$T=w_{p}X_{p}=\left[t_{1},t_{2},\ldots t_{n}\right]\in R^{s\times n},\;$$

(4) $$B=w_{t}X_{p}={\left[{b_{1}}^{T},{b_{2}}^{T},\ldots {b_{n}}^{T}\right]}^{T}\in R^{h\times n},\;$$

(5) $${e_{i}}^{j}=\frac{\exp({b_{i}}^{j})}{\sum_{s}\exp({b_{i}}^{s})},\;$$

The position-aware attention is used to update features of each ligand atom. $X_{l}\in R^{m\times c}$ represents initial drug features and the updated features can be computed by


(6) $$X_{l}^{1}=X_{l}+X_{l}⊙a_{l}^{T}.\;$$

The position-aware attention for ligand features is defined as where $w_{l}$ and $w_{g}$ are the learnable parameters and *m* is the ligand atom number. A similar process is used in protein for obtaining the updated amino acid features.


(7) $$a_{l}=\mathrm{softmax}\left({\left(w_{l}X_{l}\right)}^{T}w_{g}G^{p}\right),\;$$

#### 2.3.2 Multihead attention mechanism

In ligand atom graph, besides the general atom nodes, a virtual super node was also created to receive information from real atom nodes. Here, the multihead attention mechanism was employed to weigh the individual atom nodes contribution. For the information transfer from the general atoms to the super node, the *K*-head attention mechanism can be achieved as where *F* represents the tanh activation function, and *n* is the number of ligand atoms. $w_{a2s}$ denotes the learnable weight. In this work, we used *K *=* *4 parallel attention heads. where *i* is the number of ligand atoms and $w_{at}^{k}$ is the learnable parameters. where $w_{\mathrm{aat}}^{k}$ and $w_{\mathrm{sat}}^{k}$ are two learnable parameters. $V_{i}$ denotes the atom features, and s denotes the super node features. Furthermore, in the protein graph, the information was passed and updated by a similar process.


(8) $$I_{v2s}=F\left(w_{a2s}\left[\sum_{i=1}^{n}T_{a,i}^{1}V_{i},\;\sum_{i=1}^{n}T_{a,i}^{2}V_{i},\;\sum_{i=1}^{n}T_{a,i}^{K}V_{i}\right]\right),\;$$

(9) $$T_{a,i}^{k}=\mathrm{softmax}\left(w_{at}^{k}c_{v,i}^{k}\right),\;k=1,2,3,\;$$

(10) $$c_{v,i}^{k}=F\left(w_{\mathrm{aat}}^{k}V_{i}\right)\;*\;F\left(w_{\mathrm{sat}}^{k}s\right),\;k=1,2,3,\;$$

#### 2.3.3 Gated recurrent units

A GRU (Chung *et al.* 2014) was introduced to make each recurrent unit to capture the dependencies of different time scales. In order to update the individual atom nodes and super node features, two GRUs were used to calculate the proportion of individual atoms and super node. The super node feature’s update can be defined as and the individual atom feature’s update is computed as where ${\mathrm{tran}}_{v2s}$ represents the final transformed information from the atom nodes to super node, and ${\mathrm{tran}}_{s2v}$ represents the final transformed information from the super node to atom nodes. $I_{v2s}$, $I_{s2s}$, $I_{v2v}$, and $I_{s2v}$ stand for different types of information. $w_{s2s}$, $w_{v2s}$, $w_{v2v},$ and $w_{s2v}$ are four learnable parameters.


(11) $$s^{\prime }=\mathrm{GRU}\left(s,{\mathrm{tran}}_{v2s}\right),\;$$

(12) $${\mathrm{tran}}_{v2s}=g_{s}\;*\;I_{v2s}+\left(1-g_{s}\right)\;*\;I_{s2s},\;$$

(13) $$g_{s}=\mathrm{sigmoid}\left(w_{s2s}I_{s2s}+w_{v2s}I_{v2s}\right),\;$$

(14) $${V_{i}}^{'}=\mathrm{GRU}\left(V_{i},{\mathrm{tran}}_{s2v}\right),\;$$

(15) $${\mathrm{tran}}_{s2v}=g_{v}\;*\;I_{s2v}+\left(1-g_{v}\right)\;*\;I_{v2v},\;$$

(16) $$g_{v}=\mathrm{sigmoid}\left(w_{v2v}I_{v2v}+w_{s2v}I_{s2v}\right),\;$$

#### 2.3.4 Graph neural network

The graph neural network was constructed to extract the ligand graph features (Fig. 3B), where two types of nodes including individual atoms and a virtual super node, and one type of edge encoding were included in the ligand graph. Specifically, in total five types of information were used to calculate and update the graph node features, including (i) the atom information gathered from its neighbor atoms; (ii) super node information; (iii) transformed information from individual atoms to super node; (iv) the transformed information from super node to individual atoms; and (v) the edge information. The update of the super node features can be achieved by the tanh activation function (Supplementary Fig. S1A). The information transfer from individual atoms to super node can be achieved by the multihead attention mechanism (Supplementary Fig. S2). We can also get the transformed information from super note to individual atoms by the tanh activation function (Supplementary Fig. S1B). Furthermore, the atom information gathered from its neighbor atoms can be achieved by one-step neighbor atom’s information aggregation along the graph edges (Supplementary Fig. S3). Next, two GRUs were utilized to update the atoms and super node features, respectively. Finally, two iterations of graph neural network were used to update the ligand node features. The protein graph node features were updated by a similar graph neural network, with the only difference being that the graph edge features were not used in the graph update.

#### 2.3.5 Pocket–ligand distance-dependent graph neural network

In pocket–ligand interaction graph, a distance-based radial pooling was created to present the interaction edge. The interaction edge can be denoted as where $r_{ij}$ is the interaction distance between protein residue and ligand atom; $r_{s}$ and σ are the learnable parameters, and $f_{c}\left(r_{ij}\right)$ is defined as where $R_{C}$ is fixed to 8 Å.


(17) $$f\left(r_{ij}\right)={(\text{exp}\;}^{-\frac{{\left(r_{ij}-r_{s}\right)}^{2}}{\sigma^{2}}{)f}_{c}\left(r_{ij}\right)}),\;$$

(18) fcrij=12cos⁡πrijRC, 0<rij≤8 Å0, rij>8 Å, 

Next, the graph node features will be updated by the neighbor nodes information aggregation along different edges and the self-nodes information addition. Eventually, three iterations of distance-based radius pooling for pocket–ligand interaction graph neural network were employed to transform and update the node features.

## 3 Results

### 3.1 Model comparison

To evaluate the predictive performance of GraphscoreDTA in the protein–ligand binding affinity prediction (Supplementary Fig. S4), we compared our model with multiple existing methods on the constructed datasets. The compared methods include DeepDTA (Öztürk *et al.* 2018), DeepDTAF (Wang *et al.* 2021), Pafnucy (Stepniewska-Dziubinska *et al.* 2018), BAPA (Seo *et al.* 2021), and a fusion model (Jones *et al.* 2021). We retrained and reevaluated the prediction performance of these methods using the compound similarity-based clustering (Table 1 and Supplementary Table S3) and protein similarity-based clustering, respectively. The root mean square error (RMSE), mean absolute error (MAE), standard deviation (SD) in regression, Pearson’s correlation coefficient (*R*), and concordance index (CI) are used to measure the predictive performance. As shown in Table 1, our proposed GraphscoreDTA performs best on both datasets. The *R* values of GraphscoreDTA in the CASF2016 test set are 11.5%, 8.6%, 10.2%, 35.1%, and 11.4% larger than DeepDTA, DeepDTAF, Pafnucy, BAPA, and the fusion model. Similar improvement was also observed in the CASF2013 test set. Among remaining metrics, our model achieves best performance of 0.819 (0.039 improvement), 1.249 (reduced by 0.177), 0.981 (reduced by 0.149), and 1.216 (reduced by 0.192) for CI, RMSE, MAE, and SD, respectively. More notably, the SD metric of our model has highest drop with a value of 0.192 than other metrics, while the CI metric has the least improvement (0.039). Furthermore, we also evaluated the model performance based on protein similarity clustering, and found that our model consistently outperformed the other models in all the metrics (Table 2). These results indicate that our model can integrate the information from the global structure features and local pocket–ligand interaction features, which can help enhance our model’s ability to identify the binding affinity.

**Table 1.** Performance of GraphscoreDTA and state-of-the-art methods based on compound similarity clustering (the best results are represented in bold).

Dataset	Methods	*R*	CI	RMSE	MAE	SD
CASF2016	DeepDTA	0.745	0.771	1.458	1.177	1.461
	DeepDTAF	0.765	0.780	1.426	1.130	1.408
	Pafnucy	0.754	0.776	1.527	1.253	1.434
	BAPA	0.615	0.714	1.936	1.582	1.722
	Fusion model	0.746	0.773	1.513	1.207	1.454
	GraphscoreDTA	**0.831**	**0.819**	**1.249**	**0.981**	**1.216**
CASF2013	DeepDTA	0.710	0.754	1.608	1.319	1.612
	DeepDTAF	0.616	0.702	2.010	1.506	1.793
	Pafnucy	0.662	0.738	1.716	1.376	1.698
	BAPA	0.620	0.719	2.014	1.648	1.778
	Fusion model	0.662	0.737	1.720	1.387	1.698
	GraphscoreDTA	**0.757**	**0.782**	**1.486**	**1.179**	**1.480**

**Table 2.** Performance of GraphscoreDTA and state-of-the-art methods based on protein similarity clustering (the best results are represented in bold).

Dataset	Methods	*R*	CI	RMSE	MAE	SD
CASF2016	DeepDTA	0.728	0.762	1.578	1.264	1.581
	DeepDTAF	0.782	0.792	1.445	1.179	1.443
	Pafnucy	0.756	0.779	1.494	1.231	2.181
	BAPA	0.628	0.719	1.886	1.528	1.701
	Fusion model	0.757	0.781	1.467	1.170	1.427
	GraphscoreDTA	**0.810**	**0.811**	**1.349**	**1.053**	**1.281**
CASF2013	DeepDTA	0.654	0.731	1.785	1.417	1.790
	DeepDTAF	0.672	0.732	1.791	1.514	1.780
	Pafnucy	0.687	0.748	1.666	1.355	2.242
	BAPA	0.625	0.721	1.923	1.571	1.769
	Fusion model	0.741	0.777	1.554	1.252	1.523
	GraphscoreDTA	**0.758**	**0.780**	**1.542**	**1.235**	**1.479**

### 3.2 Model ablation

The ablation studies were conducted to evaluate the effectiveness of main strategies adopted in the modeling (Supplementary Table S4). Here, seven different ablation tests were conducted. Model I directly uses the initial features of amino acid of the protein and atom of the ligand as the input of the graph neural network, while ignoring the mutual information between proteins and ligands. Model II just uses the distance-based graph neural network (interaction information), while the mutual information, protein graph neural network and ligand graph neural network are not included in the model. Model III considers both mutual information and individual protein and ligand graph information to learn the protein–ligand interaction features. For model IV, the ablation test by dropping the Vina distance terms is executed to check the model optimization. The other three models are constructed by dropping multihead attention mechanism, skip connections and GRUs, respectively. As shown in Table S4, the GraphscoreDTA performs better than the ablation models. Besides, model II performs worse than other ablation models, indicating that individual protein and ligand GNNs have advantage to recognize the protein–ligand binding affinity. Furthermore, model III and IV show similar performance. However, model III has higher values of RMSE and MAE, while model IV has lower values of *R*, CI, and higher value of SD. We consider that model IV (Vina optimization terms) may be more effective than model III (protein–ligand interaction GNNs) for affinity prediction. The ablution studies show that combining different configurations, such as the mutual information, individual protein and ligand GNNs, interaction GNNs, and Vina optimization terms, can indeed improve the prediction performance.

### 3.3 The effect of protein contact map

Considering the information transfer between different nodes of protein graph, we used a contact map to represent the edges in a protein graph. Here, we calculated the contact maps using different distance cutoffs, then the models with different contact maps were evaluated on the CASF2016 test set. As shown in Supplementary Fig. S5A, GraphscoreDTA with the cutoff of 8 Å performs better than the cutoff of 6 and 10 Å. More specifically, the model with the cutoff of 10 Å has better performance than the cutoff of 6 Å. We speculate that the distance cutoff is related to the noncovalent interactions. Besides, we also tested the performance of GraphscoreDTA using different cutoffs pocket–ligand interaction GNNs (distance-based GNNs) on the CASF2016 test set. Interestingly, the model with the cutoff of 10 Å in the interaction GNNs performs better than the cutoff of 6 Å (Supplementary Fig. S5B). The results imply that when the cutoff value is too small, the interaction GNNs perform poorly as it may not capture long-range protein–ligand interactions.

### 3.4 Attention contribution and visualization

**Figure 4.**
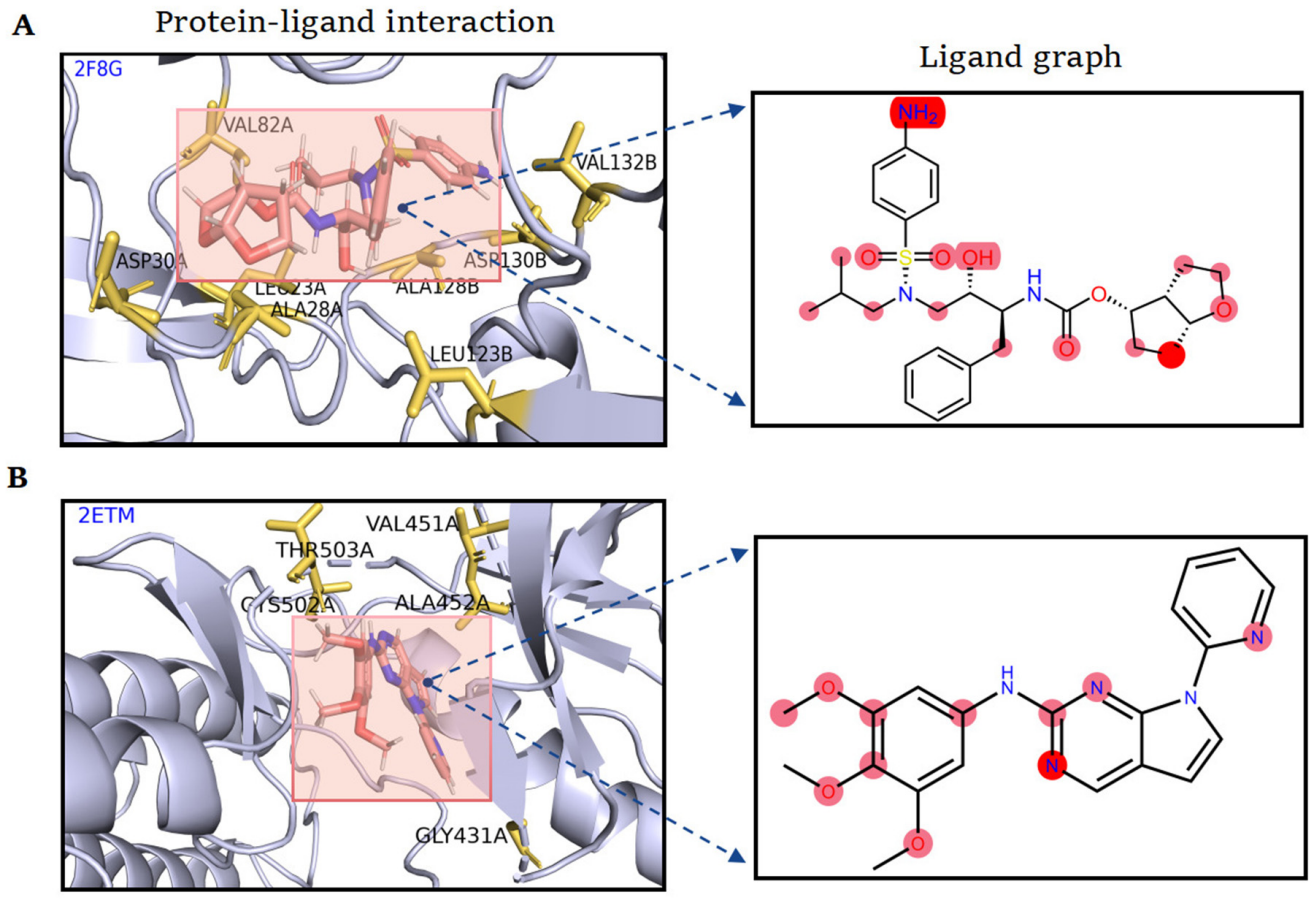
Attention visualization and contribution for protein–ligand binding affinity. (A) 2F8G. (B) 2ETM. *Left*: predicted important residues and ligand are marked with yellow and pink. Local protein structures are presented as light blue. *Right:* ligands are represented by 2D Kekule formula. The highlighted atoms are indicted the corresponding predicted important atoms

Here, two representative protein–ligand complexes were selected to visualize the attention contribution of our constructed model. The model can produce multihead attentions by inputting protein contact maps and ligand graphs, where the weights represent the contribution of each amino acid of the protein and each atom of the ligand. Thus, the amino acid/atom with a larger weight value indicates that it is more important for affinity identification. The top-15 weighted residues and atoms were selected to check the overlap with the actual interaction sites (Fig. 6; Supplementary Table S5). In HIV-1 protease mutant complexed with inhibitor TMC114 (PDB ID: 2F8G) (Kovalevsky *et al.* 2006), eight residues in the binding pocket, including LEU23A, ALA28A, ASP30A, VAL82A, LEU123B, ALA128B, ASP130B, and VAL132B (yellow), are successfully predicted. Especially, ASP30A and ASP130B can form hydrogen interactions with O26 and N1 atoms (red) of TMC114, respectively, and the other residues can form hydrophobic interactions with remain atoms (pink) of the compound, which can be recognized by our model (Fig. 4A). For Focal adhesion kinase domain complexed with 7H-Pyrrolo pyrimidine derivative (PDB ID: 2ETM), GraphscoreDTA can successfully identify five binding residues, including GLY431A, VAL451A, ALA452A, CYS502A, and THR503A (yellow). The hydrogen interaction between CYS502A and NAC atom (red) of 7PY, hydrophobic interactions between remaining highlighted residues and atoms (pink) of the compound can be correctly captured (Fig. 4B). These results show that our model can learn and highlight the important residues and atoms for proteins and ligands, which contribute to the interpretation of recognition mechanisms among protein–ligand pairs.

### 3.5 Model performance without experimental tertiary structures

Proteins possess specific structures for different folding recognition. In this study, the tertiary structures were needed for identifying the protein–ligand binding affinity which might not be always available. Here, we evaluated the model performance using the predicted structures rather than the experimental structures on the CASF2016 test set. To avoid the sequence error, protein sequences in UniProt database must completely cover the sequence parsed from the PDB dataset. Then AlphaFold2 (Jumper *et al.* 2021) was employed to predict the tertiary structures using the filtered sequences, resulting in a total of 140 structures. The predicted structures were aligned to their native structures. The aligned structures can form complexes with the originally paired small molecules, which were used to construct the pocket–ligand interaction graphs. Next, RMSDs between the crystal and predicted structures as well as RMSEs of binding affinity prediction for the 140 predicted structures were calculated. Subsequently, these structures were divided into 10 groups according to RMSD values (Supplementary Table S6 and Fig. S6). In these groups, the Pearson correlation coefficient between RMSDs and RMSEs is 0.554, suggesting that accurate modeling structure leads to better performance of binding affinity prediction. What’s more, we find that the RMSE values for most of predicted structures are ∼1, especially for the structures with RMSD values <1.1 Å, which further demonstrates the positive correlation between accuracy of modeling structures and accuracy for affinity prediction. It is thus not surprising that the RMSE values become significantly larger when the RMSD values are >15 Å. While the aligned structures are also not available when the experimental structures are not available. Hence we also constructed the pocket–ligand interaction graphs based on docked structures with lowest-scoring conformations of ligands bound to predicted proteins using AutoDock Vina. It is noted that our model based on the docked conformations and the aligned protein–ligand structures achieves an average RMSE value of 1.010 and 1.008, respectively. Furthermore, the RMSE values based on docked conformations and aligned protein–ligand structures have the same Pearson correlation coefficient (0.554) with the RMSD values between the predicted protein structures and their native structures, suggesting that GraphscoreDTA has similar predictions for the docked conformations and the aligned protein–ligand structures. All these results indicate that the modeling structures with RMSD values <1.1 Å can be helpful to predict the binding affinity when the number of experimentally solved protein structures is insufficient.

### 3.6 Model performance for cyclin-dependent kinases

The cyclin-dependent kinase (CDK) family plays a vital role in the regulation of the eukaryotic cell cycle and has important functions in apoptosis, transcription, and differentiation (Malumbres 2014). Cyclin-dependent kinase 9 (CDK9), a kinase of positive transcription elongation factor, is an important validated target for the treatment of diseases, including cardiac hypertrophy, cancer and human immunodeficiency virus. Over the past two decades, multiple CDK inhibitors have been developed. Here, we collected all the structures of CDK9 in complexes with different inhibitors from the datasets. A total of 11 complexes were collected to evaluate the model performance. As expected, GraphscoreDTA achieves high prediction accuracy, the *R*, CI, RMSE, MAE, and SD values are 0.876, 0.704, 0.470, 0.376, and 0.484, respectively. The results suggest that the predicted values are extremely close to the measured values (Supplementary Fig. S7). For example, flavopiridol is an anticancer drug in phase II clinical trials (Baumli *et al.* 2008), which can bind to the ATP site of CDK9 inducing structural changes (PDB ID: 3BLR). GraphscoreDTA gives the predicted value of 7.741, which is close to the measured value (8.19).

**Figure 5.**
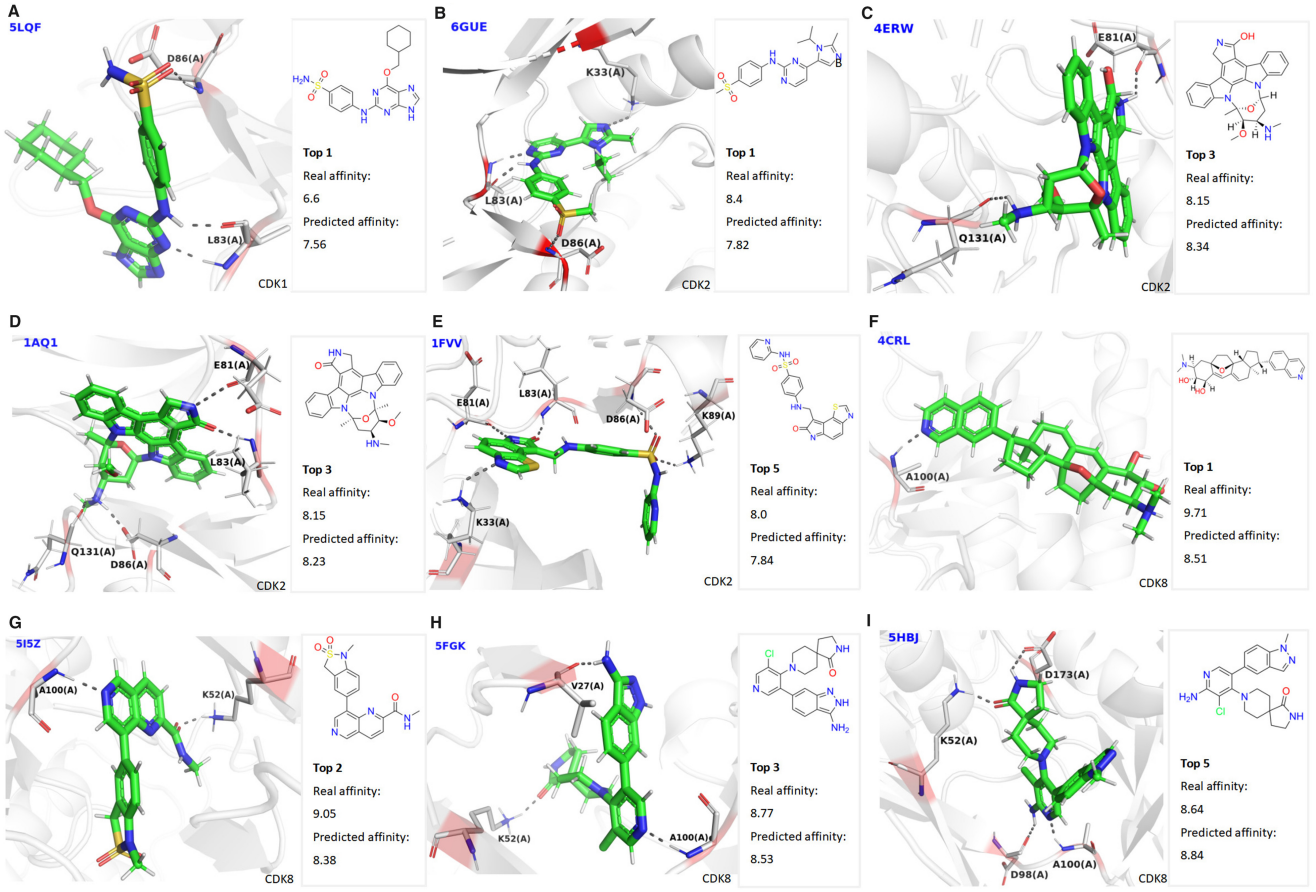
The interactions and binding affinity values between the compounds and the selected kinases targets

Furthermore, we evaluated the performance on the other CDKs including CDK1, CDK2, and CDK8. We collected 5 crystal structures of CDK1 in complexes with compounds, 77 crystal structures of CDK2 in complexes with compounds, and 21 crystal structures of CDK8 in complexes with compounds from the constructed datasets. We calculated the total number of hits among the top 1, top 10, and top 8 predicted candidate compounds for CDK1, CDK2, and CDK8, respectively. More specifically, the top 1 of the known compounds with best measured binding affinity value for CDK1 target was correctly predicted as top ranked by our model (Fig. 5A). Meanwhile, the top 1 (Fig. 5B), two top 3 (Fig. 5C and D), and top 5 (Fig. 5E) of the known compounds with best measured binding affinity values for CDK2 target were correctly predicted among the top 10 predicted using our model. Moreover, the top 1 (Fig. 5F), top 2 (Fig. 5G), top 3 (Fig. 5H), and top 5 (Fig. 5I) of the known compounds with best measured binding affinity values for CDK8 target were also correctly predicted among the top 8 predicted by our model. According to the measured values, it is known that o6-cyclohexylmethoxy-2-(4′-sulphamoylanilino) purine (Fig. 5A), 4-(2-methyl-3-propan-2-yl-imidazol-4-yl)-∼{N}-(4-methylsulfonylphenyl) pyrimidin-2-amine (Fig. 5B), and cortistatin A (Fig. 5F) maintain the highest affinity values against other compounds with CDK1, CDK2, and CDK8, respectively. As expected, our model can successfully hit them. The recommendation results were also compared between our method and other models (Supplementary Table S7). The results indicate that our model can identify the largest number of compounds among all the methods. The names of 9 compounds and their targets are summarized in Supplementary Table S8.

### 3.7 Predicting target selectivity of drugs

We applied GraphscoreDTA to the task of drug–target selectivity. Here, the drug–targets with large differences in binding affinity values were considered to evaluate the model performance. For drug catechol, we aim to find the effective targets with the highest pairwise binding affinities (Supplementary Table S9). It is known that the neutrophil gelatinase-associated lipocalin protein (Bao *et al.* 2010) (PDB ID: 3FW4) maintains the highest affinity value against other protein targets with this compound. GraphscoreDTA successfully predicted this target at the top. Furthermore, we also choose the drug–targets with similar binding affinity values to evaluate the model performance (Supplementary Table S10). For drug (5R,6R,7S,8R)-5-(hydroxymethyl)-5,6,7,8-tetrahydroimidazo[1,2-a]pyridine-6,7,8-triol, GraphscoreDTA can also successfully predict the top target. In comparison, the RMSE value (1.40) of GraphscoreDTA is smaller than the value (2.38) of DeepDTAF for the first-ranked target of catechol. Furthermore, for (5R,6R,7S,8R)-5-(hydroxymethyl)-5,6,7,8-tetrahydroimidazo[1,2-a]pyridine-6,7,8-triol, the RMSE values of GraphscoreDTA for the first-ranked and second-ranked targets are 0.60, 0.20, respectively, which are 13.0% and 89.2% lower than that of Pafnucy. The results demonstrate that GraghscoreDTA has a promising performance to predict the target selectivity of the specific drugs.

### 3.8 Drug–target selectivity of homologous protein family

**Figure 6.**
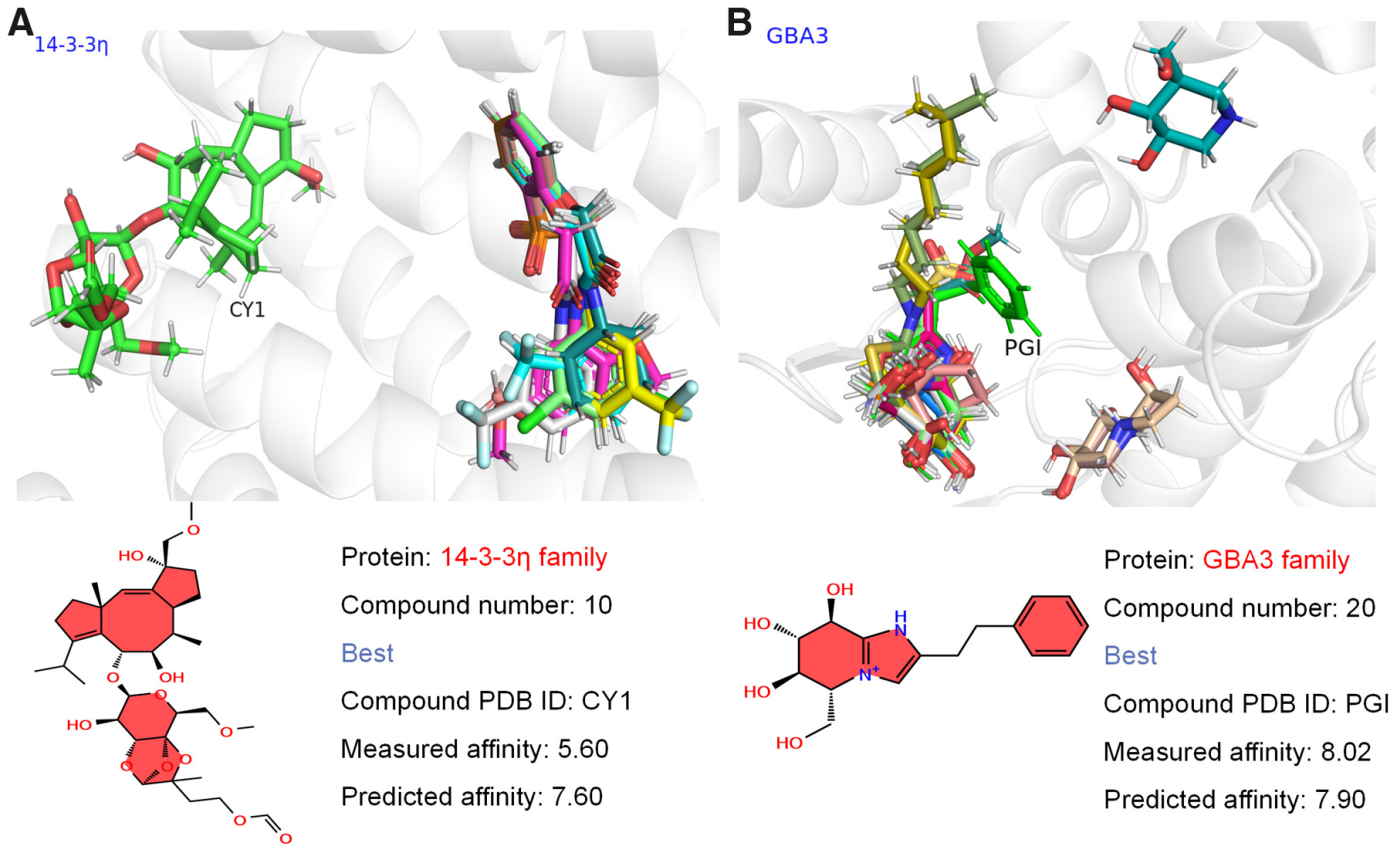
CY1 and PGI with the highest experimental binding affinity values in the 14-3-3η (A) and GBA3 (B) families can be successfully identified by GraphscoreDTA

A total of 8 homologous proteins with 191 ligands were chosen from the constructed datasets to evaluate the model performance, including 14-3-3 protein (14-3-3*η*) with 10 ligands, 3-dehydroquinate-dependent protein kinases-1 (DHQD) with 19 ligands, 3-phosphoinositide-dependent protein kinase-1 (PDPK1) with 25 ligands, acetylcholine receptor (AChR) with 29 ligands, *β*-glucosidase (GBA3) with 20 ligands, biotin carboxylase (BC) with 10 ligands, protein kinase A (PKA) with 45 ligands, and dual-specificity phosphatase (DSP) with 33 ligands (Supplementary Table S11). GraphscoreDTA successfully predicted 14-3-3 in complex with Cotylenin A (PDB ID: 3E6Y) and GBA3 from Thermotoga maritima in complex with phenethyl-substituted glucoimidazole (PDB ID: 2CET), both of which have the highest experimental binding affinity values against the other 9 and 19 compounds in the 14-3-3*η* and GBA3 families, respectively (Fig. 6). Meanwhile, the top interactions in AChR family MACHE-Y337A mutant in complex with soaked TZ2PA6 SYN inhibitor (PDB ID: 2XUP) can be predicted as top 2 using our model. Furthermore, the inhibitors LZK, CB6, 796, ABQ, and EUI are highly selective toward the proteins (PDB IDs: 2V59, 2Y71, 2GU8, 3RWP, and 4LMN) in BC, DHQD, PKA, PDPK1, and DSP families, which can be accurately identified in the top 3, top 4, top 4, top 6, and top 7 using GraphscoreDTA, respectively. Moreover, GraphscoreDTA outperformed the other methods for most of the protein sets (Supplementary Fig. S8). For the 14-3-3η protein set, the MAE value of GraphscoreDTA is relatively poor (2.242), most likely due to its special alpha-helix secondary structure, but GraphscoreDTA shows good drug selectivity for 14-3-3η protein set.

## 4 Discussion

Determination of protein–ligand binding affinity is a challenging task in drug discovery and development. In this paper, a graph neural network-based architecture with the Vina distance optimization terms was developed to predict the protein–ligand binding affinity. Different from traditional graph neural networks, five strategies, such as bitransport information mechanism, skip connection, multihead attention, GRUs and the Vina distance optimization terms, were employed in this architecture. Extensive analyses suggest that GraphscoreDTA is a promising tool to identify the target selectivity toward the drugs and the drug selectivity toward the targets.

In addition, the protein pocket possesses special properties to bind the compound. To further validate the significance of binding pocket, we attempted to explore the relationship between pocket and protein–ligand interaction. For example, D0PY27 (UniPort ID), a potential target, can bind different small molecules and play a relevant role in the treatment of Hepacivirus’s disease. It is interesting to note that the sizes of pocket and small molecules relate to the protein–ligand binding affinity (Supplementary Fig. S9). Therefore, integrating the sizes of pocket and small molecules may enhance the prediction accuracy. In the future, we will consider it in the protein–ligand interaction prediction task. The improvement over the regression prediction indicates that the new strategy may contribute to the 3D structure-based predictions, such as protein–protein interaction, protein–RNA interaction, RNA–ligand interaction (Wang *et al.* 2018, 2023), and drug–drug interaction (Zagidullin *et al.* 2021, Zheng *et al.* 2021).

## Supplementary Material

btad340_Supplementary_Data

## Data Availability

The data underlying this article are available in the PDBbind database, at http://www.pdbbind.org.cn/.
