# Supplementary material for: GraphscoreDTA: optimized graph neural network for protein–ligand binding affinity prediction
Source: Bioinformatics. 2023 May 24;39(6):btad340. doi: 10.1093/bioinformatics/btad340 (PMC10243863; doi:10.1093/bioinformatics/btad340)
Supplement: btad340_Supplementary_Data [file btad340_supplementary_data.zip › GraphscoreDTA_supplementary_bio.docx]

GraphscoreDTA: optimized graph neural network for protein-ligand binding affinity prediction

Kaili Wang^1^, Renyi Zhou^1^, Jing Tang^2,3^, Min Li^1,4*^

^1^School of Computer Science and Engineering, Central South University, Changsha 410083, China

^2^Institute for Molecular Medicine Finland (FIMM), University of Helsinki, 00014 Helsinki, Finland

^3^Department of Biochemistry and Developmental Biology, University of Helsinki, 00014 Helsinki, Finland

^4^Hunan Provincial Engineering Research Center of Intelligent Computing in Biology and Medicine, Changsha 410083, China

***Corresponding authors**

**Min Li**

**Email: limin@mail.csu.edu.cn**

Section S1

1. **Protein representation**

In our architecture, the protein 3D structures were transformed into graphs, in which the amino acids in the given protein were defined as nodes and two nonconsecutive amino acids were connected by an edge if they contain a pair of Cα atoms, one from each amino acid and less than 8 Å apart.

1.1 Node feature matrix

Here, one group of sequence features, namely, primary sequence information, normalized position information and evolutionary relationship information, were concatenated and employed to represent node feature matrix.

***Primary sequence information.*** Amino acids are the components of protein sequences. Here, we used a 20D one-hot vector to encode amino acid types in a specific protein sequence.

***Normalized position information.*** The position of an amino acid in a protein sequence is divided by the sequence length.

***Evolutionary relationship information.*** BLOSUM62 matrix is a 20×20 matrix that contains the evolutionary relationships between amino acids. In this study, a protein sequence was encoded by BLOSUM62 matrix, in which the column of BLOSUM62 matrix was used to represent specific amino acid type. Therefore, 20D vector was encoded to represent the evolutionary relationship information.

In summary, a 41D sequence-based feature vector, including primary sequence information, normalized position information, and evolutionary relationship information, was employed to denote the graph node features.

1.2 Adjacency matrix

Considering the connectivity of graph nodes, calculating an adjacency matrix is necessary for representing the edges in a protein graph. In a specific graph, the amino acids were defined as nodes. Besides, the non-covalent interactions between amino acids were defined as edges. More specifically, two nonconsecutive amino acids were connected as an edge if the Euclidean distance of Cα atom pair for any two amino acids is less than 8 Å. Then, the distances between all amino acids pairs in one protein can form a distance map. The distance map was further transformed into an adjacency matrix by reassigning into any edge value less than or equal to 8 Å to 1, and any edge value larger than 8 Å to 0.

**2 Ligand representation**

In the compound graph, nodes denote atoms and edges represent chemical bonds, which can be processed by RDKit. More specifically, the initial node features of ligand were represented by the 63D one-hot encoding of atom type, 6D one-hot encoding of atom degree, 6D one-hot encoding of atom explicit valence, 6D one-hot encoding of atom implicit valence, and 1D feature encoding of atom aromaticity. Finally, an 82-dimensional feature vector was created to represent the initial node features. In the representation of graph edges, six bond types were employed. Here, the edge types consist of single-bond, double-bond, triple-bond, aromatic-bond, conjugated, and ring. Therefore, a 12D feature vector was created to encode the edge information.

**3 Interaction representation**

An interaction-based subgraph was constructed to represent pocket-ligand interaction information. Here, we defined the amino acids of proteins and the atoms of ligands as interaction node pairs. Specifically, we employed a 41D sequence-based feature vector to denote amino acid node features. Besides, an 82D atom feature vector employed in ligand representation was created to represent atom node features. The two nodes, namely, amino acid of the protein and atom of the ligand, are connected by an edge if the Euclidean distance between at least one pair of heavy atoms is less than 8 Å. Furthermore, the edge feature is defined as ${edge}_{inter}={(8 Å-distance)}/{8 Å}$ if the distance is less than 8 Å, else 0.

Section S2

1. **Vina distance optimization terms**

In our study, the defined Vina distance optimization terms inherit the same weighted scoring terms from AutoDock Vina. More specifically, the Vina distance optimization terms are made up of five docking conformation-dependent terms and one conformation-independent term. The five conformation-dependent terms count for intermolecular contributions, which include three terms accounted for steric interactions, one hydrophobic bond term, and one hydrogen bond term. The conformation-independent term counts for flexible contribution, which is the number of active rotatable bonds $N_{rot}$ between heavy atoms of the ligand. The six terms are defined as follows:

$$\begin{aligned} {Gauss}_{1}\left( t_{i},t_{j},r_{ij} \right)={w_{1}e}^{{-\left( {d_{ij}}/{0.5} \right)}^{2}}\#\left( \text{1} \right) \end{aligned}$$

$$\begin{aligned} {Gauss}_{2}\left( t_{i},t_{j},r_{ij} \right)={w_{2}e}^{{-((d_{ij}-3)/2)}^{2}}\#\left( \text{2} \right) \end{aligned}$$

$Repulsion\left( t_{i},t_{j},r_{ij} \right)=\left\{ \begin{matrix} {w_{3}d}_{ij}^{2} & if d_{ij}<0 \\ 0 & if d_{ij}\geq0 \end{matrix} \right.$ (3)

$Hydrophobic\left( t_{i},t_{j},r_{ij} \right)=\left\{ \begin{matrix} w_{4} & if d_{ij}\leq0.5 \\ w_{4}\left( 1.5-d_{ij} \right) & if 0.5<d_{ij}<1.5 \\ 0 & d_{ij}\geq1.5 \end{matrix} \right.$ (4)

$HBonding\left( t_{i},t_{j},r_{ij} \right)=\left\{ \begin{matrix} w_{5} & if d_{ij}\leq-0.7 \\ w_{5}\left( {d_{ij}}/\left( -0.7 \right) \right) & if-0.7<d_{ij}<0 \\ 0 & if d_{ij}\geq0 \end{matrix} \right.$ (5)

$Flexibility=w_{6}N_{rot}$ (6)

where the surface distance $d_{ij}=r_{ij}-R_{t_{i}}-R_{t_{j}}$. $t_{i}$ and $t_{j}$ are the atom types of *i* and *j*, respectively, excluding hydrogen atom. $R_{t_{i}}$ and $R_{t_{j}}$ are the Van der Waals radii of $t_{i}$ and $t_{j}$, respectively. $r_{ij}$ is their interatomic distance with the cutoff at $r_{ij}=8 Å$. All the distances are the Euclidean distances and all the units are in $Å$. The weights are derived from AutoDock Vina, $w_{1}$,$w_{2}$,$w_{3}$,$w_{4}$,$w_{5}$, and $w_{6}$ are -0.0356, -0.00516, 0.840, -0.0351, -0.587, and 0.0585, respectively.

**
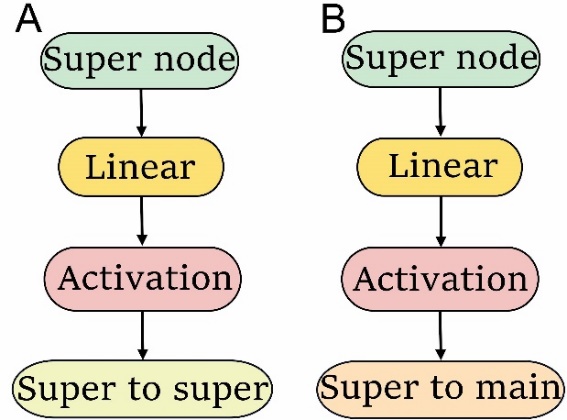
**

**Supplementary Figure S1.** Information transfer of the super node.

**
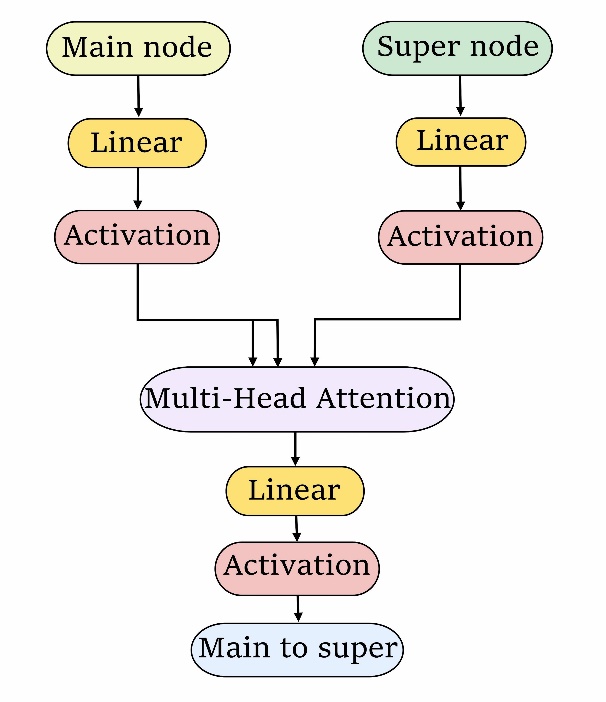
**

**Supplementary Figure S2.** Information transfer of main nodes to super node.


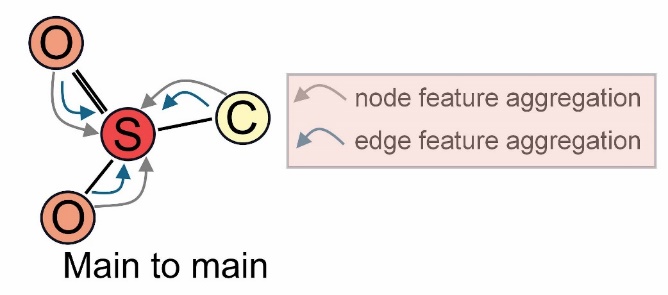


**Supplementary Figure S3.** Atom information gathered from its neighbor atoms.


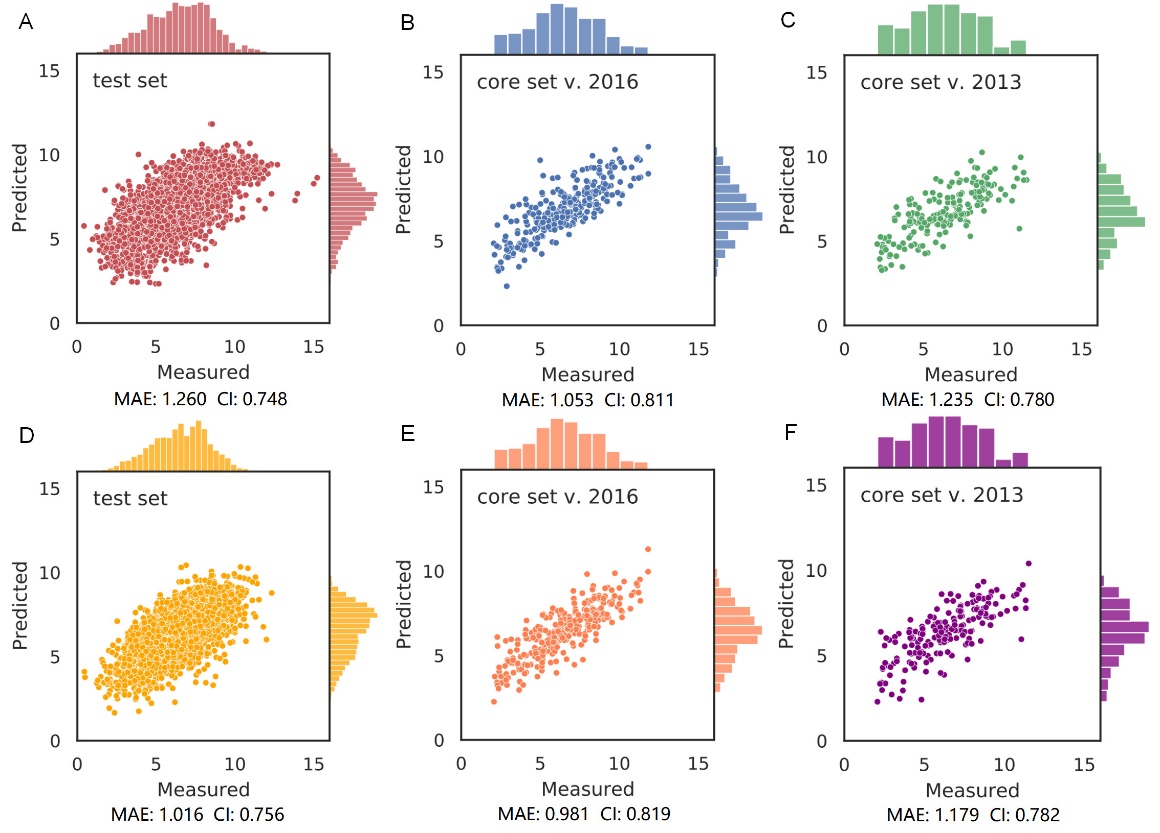


**Supplementary Figure S4.** Predictions based on protein/compound similarity clustering on PDBbind (A/D), CASF2016 (B/E) and CASF2013 (C/F) test sets.


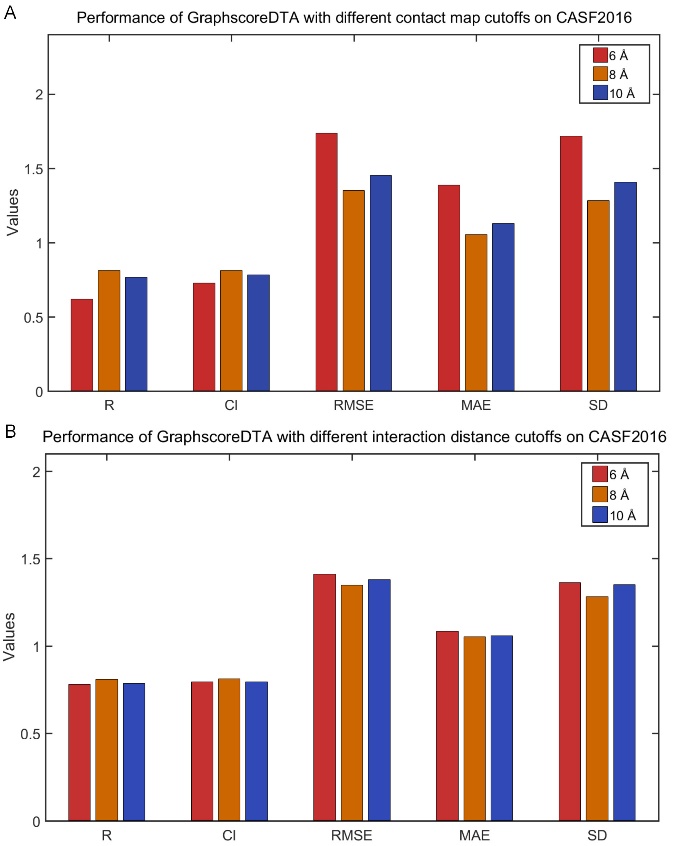


**Supplementary Figure S5.** Model performances with different contact map cutoffs (A) and different protein-ligand interaction distance cutoffs (B) on CASF2016 test set.

**
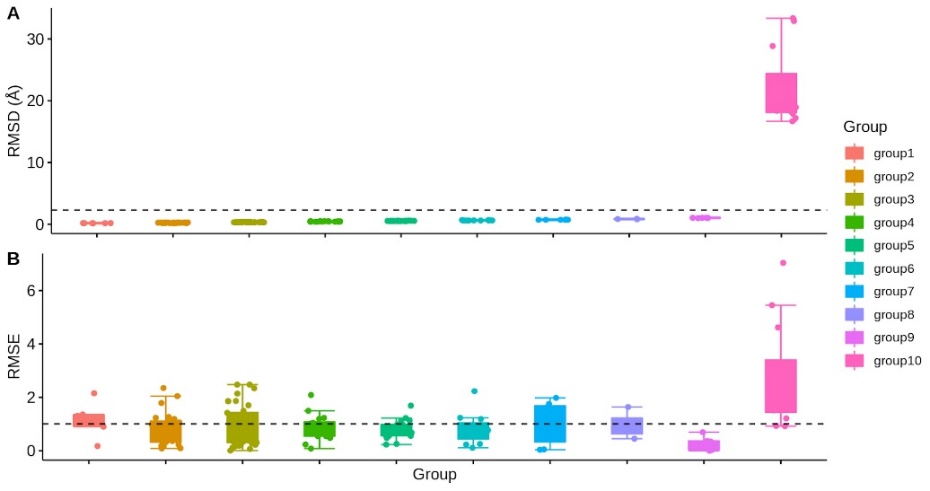
**

**Supplementary Figure S6.** Correlation between RMSD (A) and RMSE (B) in 10 groups.


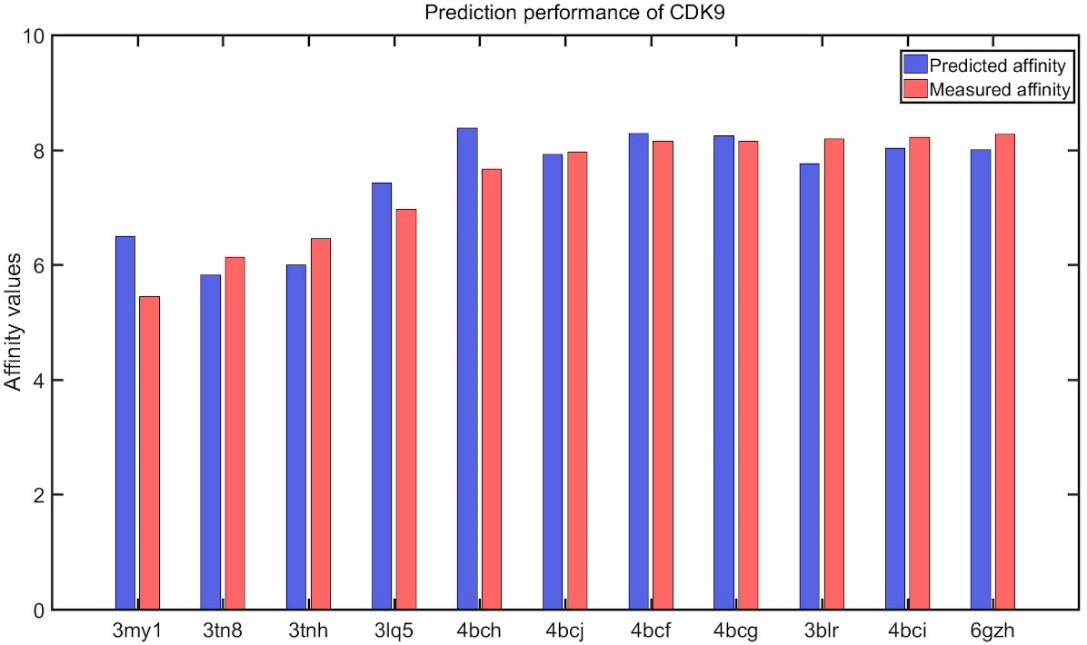


**Supplementary Figure S7.** Model performance of CDK9 in complexes with different inhibitors from the whole data.


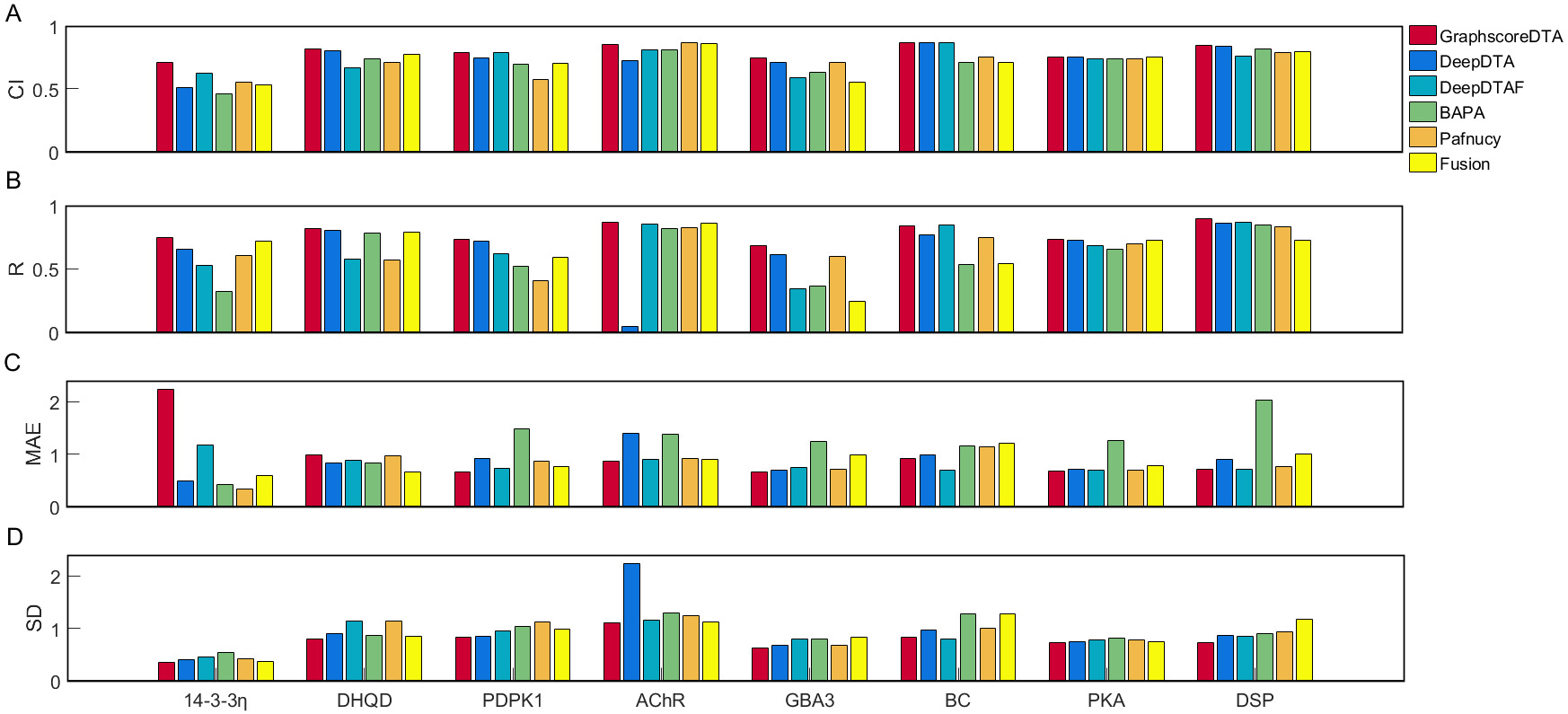


**Supplementary Figure S8.** Model performances for selected homologous protein families.


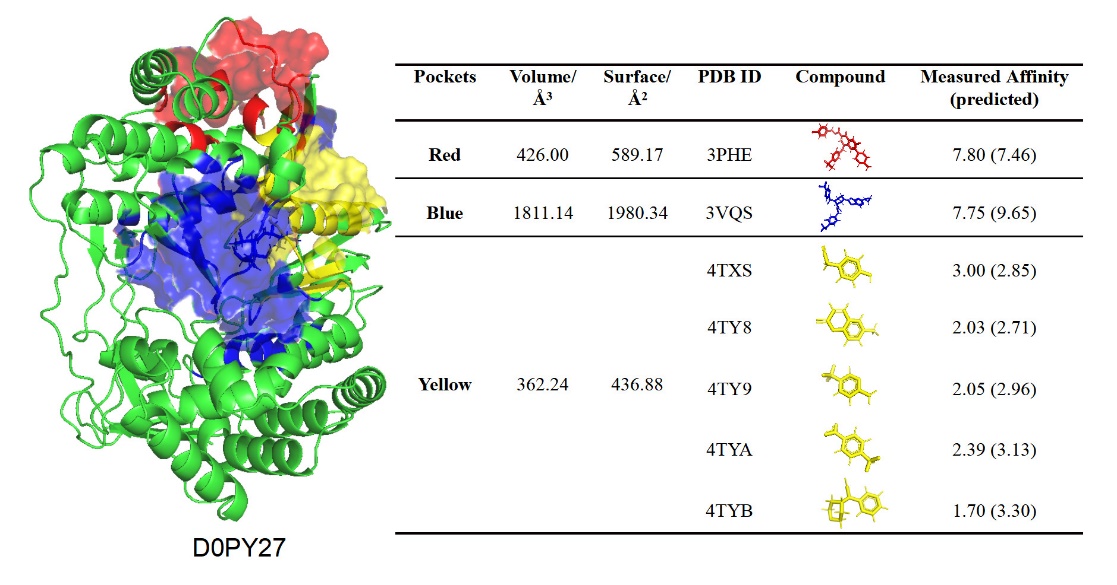


**Supplementary Figure S9.** Pocket representation of D0PY27 and the corresponding affinity values with different compounds.

**Supplementary Table S1.** Summary of the training, validation and test sets

| Clustering | Training set | Validation set | Test set |
| --- | --- | --- | --- |
| Compound similarity clustering | 6471 clusters (9869 complexes) | 878 clusters  (1328 complexes) | 1756 clusters  (2654 complexes) |
| Protein similarity clustering | 1730 clusters  (9674 complexes) | 247 clusters  (1158 complexes) | 494 clusters  (3019 complexes) |

**Supplementary Table S2.** Hyperparameter settings for GraphscoreDTA model

| Blocks | Hyperparameters | Setting |
| --- | --- | --- |
|  | Epoch | 80 |
|  | Batch size | 8 |
|  | Optimizer | AdamW |
|  | Learning rate | 1.2e-4 |
|  | Weight decay | 1e-6 |
| Ligand GNN block | Iteration | 2 |
|  | Graph node feature size | 120 |
|  | Hidden size (node) | 120 |
|  | Graph edge feature size | 8 |
|  | Attention heads | 4 |
|  | Activation function | LeakyReLU, Tanh, Sigmoid |
| Protein GNN block | Iteration | 2 |
|  | Graph node feature size | 120 |
|  | Hidden size (node) | 120 |
|  | Attention heads | 4 |
|  | Activation function | LeakyReLU, Tanh, Sigmoid |
| Pocket-ligand interaction distance-based GNN block | Iteration | 1 |
|  | Graph node feature size | 120 |
|  | Distance cutoff | 8 Å |
|  | Depth | 3 |
|  | Activation function | ReLU |
| Fully connected neural network block | Number of neurons | [366, 298, 160] |
|  | Activation function | PReLU |

**Supplementary Table S3.** Performance comparison of GraphscoreDTA with state-of-the-art methods on the PDBbind test set based on compound similarity clustering

| Methods | R | CI | RMSE | MAE | SD |
| --- | --- | --- | --- | --- | --- |
| DeepDTA | 0.670 | 0.739 | 1.390 | 1.089 | 1.390 |
| DeepDTAF | 0.675 | 0.744 | 1.369 | 1.062 | 1.359 |
| Pafnucy | 0.660 | 0.734 | 1.403 | 1.094 | 1.385 |
| BAPA | 0.526 | 0.680 | 1.643 | 1.319 | 1.567 |
| Fusion model | 0.647 | 0.731 | 1.437 | 1.123 | 1.405 |
| GraphscoreDTA | **0.708** | **0.756** | **1.315** | **1.016** | **1.300** |

*Note:* The best results are represented in bold.

**Supplementary Table S4.** Ablation studies on all metrics for our proposed model on CASF2016 test set

| Methods | R | CI | RMSE | MAE | SD |
| --- | --- | --- | --- | --- | --- |
| Without mutual information | 0.763 | 0.789 | 1.427 | 1.089 | 1.411 |
| Distance-based GNNs (without individual protein (compound) GNNs & mutual information) | 0.648 | 0.730 | 1.743 | 1.391 | 1.663 |
| Without Distance-based GNNs | 0.757 | 0.780 | 1.485 | 1.168 | 1.428 |
| Without Vina distance terms | 0.749 | 0.776 | 1.477 | 1.164 | 1.448 |
| Without Multi-head attention | 0.761 | 0.781 | 1.453 | 1.139 | 1.416 |
| Without skip connections | 0.782 | 0.796 | 1.421 | 1.105 | 1.362 |
| Without GRUs | 0.785 | 0.797 | 1.372 | 1.084 | 1.353 |
| GraphscoreDTA | **0.810** | **0.811** | **1.349** | **1.053** | **1.281** |

*Note:* The best results are represented in bold.

**Supplementary Table S5.** The top-15 weighted residues in protein 2F8G and 2ETM

| PDB ID | Residues |
| --- | --- |
| 2F8G | ALA22A, LEU23A, ALA28A, ASP30A, THR31A, VAL82A, ASN83A, ILE84A, LEU123B, LEU124B, ALA128B, ASP130B, VAL132B, ILE133B, VAL177B |
| 2ETM | ILE422A, GLY431A, GLY439A, LEU449A, VAL451A, ALA452A, LEU486A, CYS502A, THR503A, SER539A, ALA548A, ASN551A, ASP558A, VAL560A, GLY566A |

**Supplementary Table S6.** These structures are divided into 10 groups according to RMSD values between crystal and real structures

| Groups | RMSD (Å) | PDB ID |
| --- | --- | --- |
| Group1 | 0.1 < RMSD <= 0.2 | 3D4Z, 1S38, 3GY4, 3DX1, 4KZ6, 3EJR |
| Group2 | 0.2 < RMSD <= 0.3 | 1UTO, 3RYJ, 3GR2, 2C3I, 3FCQ, 1O3F, 3GV9, 1K1I, 4JXS, 4K18, 1H22, 2J78, 3D6Q, 3PWW, 1H23, 4ABG, 1GPN, 2CBV, 3JYA, 3PRS, 3OE5, 2R9W, 1R5Y, 4DJV, 3WZ8, 3NW9, 2V00 |
| Group3 | 0.3 < RMSD <= 0.4 | 3ACW, 1W4O, 2WCA, 2QBQ, 2BRB, 3TSK, 3TWP, 2J7H, 3BGZ, 1Z9G, 4GR0, 1QF1, 4AGQ, 2HB1, 3EHY, 2WEG, 2CET, 3NQ9, 4CRA, 3OZS, 3OE4, 2QBR, 5DWR, 3QQS, 4CRC, 1U1B, 1O0H, 2VVN, 4AGP, 1BZC, 4GID, 2W66, 2VKM, 1LPG, 3NX7, 4TY7 |
| Group4 | 0.4 < RMSD <= 0.5 | 3RSX, 1O5B, 4EA2, 2YKI, 4K77, 3JVS, 4OGJ, 3JVR, 1C5Z, 3UI7, 5C2H, 4IVD, 1NVQ, 2W4X, 4LLX, 4X6P |
| Group5 | 0.5 < RMSD <= 0.6 | 1OWH, 4WIV, 3U5J, 4GFM, 3G0W, 3G2N, 5C28, 1YC1, 3PYY, 3P5O, 4IVB, 1SQA, 3UUO, 4LZS, 4IVC, 2FXS, 4HGE |
| Group6 | 0.6 < RMSD <= 0.7 | 3B65, 3B5R, 4F09, 1Z95, 4M0Y, 2VW5, 3B27, 2YGE, 3RLR, 2IWX, 3B68 |
| Group7 | 0.7 < RMSD <= 0.8 | 2V7A, 4F9W, 4E6Q, 2ZCQ, 3E93, 4QD6, 4JIA |
| Group8 | 0.8 < RMSD <= 0.9 | 4DLI, 3E92, 2ZB1 |
| Group9 | 0.9 < RMSD <= 1.1 | 4KZQ, 4KZU, 4J3L, 4J21, 3KR8 |
| Group10 | 15 < RMSD | 4TMN, 2WBG, 3COZ, 4DDK, 1NC3, 5TMN, 4EKY, 3UEX, 3UEW, 4DDH, 1NC1, 3RR4 |

**Supplementary Table S7.** Model performances for 5 selected compounds against CDK1, 77 selected compounds against CDK2 and 21 selected compounds against CDK8

| Methods | CDK1 (5 compounds)  numbers of top1 hit top1 | CDK2 (77 compounds)  numbers of top10 hit top5 | CDK8 (21 compounds)  numbers of top8 hit top5 |
| --- | --- | --- | --- |
| DeepDTA | 0 | 2 | 2 |
| DeepDTAF | **1** (top) | 3 | 3 |
| Pafnucy | 0 | 3 | 2 |
| BAPA | 0 | 3 | 2 (top) |
| Fusion model | 0 | 0 | 2 |
| GraphscoreDTA | **1** (top) | **4** (top) | **4** (top) |

*Note:* The best results are represented in bold. The top in brackets refers to one of the hits ranked first in the measurement.

**Supplementary Table S8.** The names of compounds and targets in different PDB structures

| PDB ID | Target name | Compound name |
| --- | --- | --- |
| 5LQF | CDK1 | o6-cyclohexylmethoxy-2-(4’-sulphamoylanilino) purine |
| [6GUE](https://www.rcsb.org/structure/6GUE) | [CDK2](https://www.rcsb.org/structure/6GUE) | 4-(2-methyl-3-propan-2-yl-imidazol-4-yl)-~{N}-(4-methylsulfonylphenyl)pyrimidin-2-amine |
| 4ERW | [CDK2](https://www.rcsb.org/structure/6GUE) | Staurosporine |
| 1AQ1 | [CDK2](https://www.rcsb.org/structure/6GUE) | Staurosporine |
| 1FVV | CDK2 | 4-[(7-oxo-7h-thiazolo[5,4-e]indol-8-ylmethyl)-amino]-n-pyridin-2-yl-benzenesulfonamide |
| 4CRL | CDK8 | Cortistatin A |
| 5I5Z | CDK8 | N-methyl-8-(1-methyl-2,2-dioxo-2,3-dihydro-1H-2lambda~6~,1-benzothiazol-5-yl)-1,6-naphthyridine-2-carboxamide |
| 5FGK | CDK8 | 8-[3-(3-azanyl-2~{H}-indazol-6-yl)-5-chloranyl-pyridin-4-yl]-2,8-diazaspiro[4.5]decan-1-one |
| 5HBJ | CDK8 | 8-[2-azanyl-3-chloranyl-5-(1-methylindazol-5-yl)pyridin-4-yl]-2,8-diazaspiro[4.5]decan-1-one |

**Supplementary Table S9.** Model performances for drug catechol against three target proteins

| Protein | DeepDTA | DeepDTAF | Pafnucy | BAPA | Fusion model | GraphscoreDTA | Ground |
| --- | --- | --- | --- | --- | --- | --- | --- |
|  | catechol | catechol | catechol | catechol | catechol | catechol | catechol |
| Neutrophil gelatinase-associated lipocalin | 4.61 | **7.02** | 3.68 | 3.44 | 3.91 | **8.0** | 9.4 |
| Mopr | **5.20** | 5.32 | **3.86** | **3.65** | **4.30** | 5.94 | 5.39 |
| Peroxiredoxin-5 | 4.96 | 4.14 | 3.45 | 3.04 | 3.65 | 2.80 | 2.82 |

*Note:* The best results are represented in bold. The PDB IDs of Neutrophil gelatinase-associated lipocalin complexed with drug catechol, Mopr complexed with drug catechol, and Peroxiredoxin-5 complexed with drug catechol are 3FW4, 5KBI, and 4K7I, respectively.

**Supplementary Table S10.** Model performances for drug (5R,6R,7S,8R)-5-(hydroxymethyl)-5,6,7,8-tetrahydroimidazo[1,2-a]pyridine-6,7,8-triol against three target proteins

| Protein | DeepDTA | DeepDTAF | Pafnucy | BAPA | Fusion model | GraphscoreDTA | Ground |
| --- | --- | --- | --- | --- | --- | --- | --- |
|  | C1 | C1 | C1 | C1 | C1 | C1 | C1 |
| Mannosyl-oligosacc haride 1,2-alpha-mannosidase | 7.50 | **5.94** | **6.64** | 4.31 | 5.55 | **6.73** | 7.33 |
| Putative alpha-1,2-mannosidase | **7.67** | 4.11 | 4.55 | **5.33** | 5.44 | 6.60 | 6.4 |
| Beta-mannosidase | 6.43 | 4.86 | 5.41 | 4.31 | **7.32** | 5.66 | 5.85 |

*Note:* The best results are represented in bold. C1 refers to compound (5R,6R,7S,8R)-5-(hydroxymethyl)-5,6,7,8-tetrahydroimidazo[1,2-a]pyridine-6,7,8-triol. The PDB IDs of Mannosyl-oligosacc haride 1,2-alpha-mannosidase complexed with drug C1, Putative alpha-1,2-mannosidase complexed with drug C1, and Beta-mannosidase complexed with drug C1 are 4AYQ, 6F92, and 2VMF, respectively.

**Supplementary Table S11.** List of PDB ID’s included in the 8 homologous protein families

| Name | Abbreviation | Ligand Count | PDB ID |
| --- | --- | --- | --- |
| 14-3-3 protein | 14-3-3η | 10 | 4DHR, 4DHS, 4DHP, 3T0M, 4DHQ, 4DHM, 3T0L, 4DHT, 4DHO, 3E6Y |
| 3-dehydroquinate dehydratase | DHQD | 19 | 4B6O, 1V1J, 4B6P, 4B6R, 4IUO, 1GU1, 4GUJ, 3N87, 2C4W, 4CIV, 2XD9, 2WKS, 2Y71, 2Y77, 2C57, 2XB9, 2XDA, 2Y76, 3N7A |
| 3-phosphoinositide-dependent protein kinase-1 | PDPK1 | 25 | 3QD0, 2R7B, 3QD3, 3RWP, 3H9O, 3IOP, 3QCS, 1UU7, 3QD4, 3HRF, 4RQK, 3QCQ, 2XCK, 3QCX, 3RWQ, 1UU8, 1UVR, 3ION, 3QCY, 2PE1, 1Z5M, 2PE2, 1UU9, 2XCH, 3RCJ |
| Acetylcholine receptor | AChR | 29 | 2PGZ, 1GQS, 1VOT, 1QON, 2W8G, 2CKM, 2CMF, 1W76, 2W8F, 2J3Q, 2PH9, 1U65, 2WNJ, 1UW6, 2HA6, 2HA4, 2HA3, 1N5R, 2HA2, 2HA5, 2XUP, 2HA7, 1Q84, 1UV6, 2XYS, 1GPK, 2WNC, 1H23, 1E66 |
| Beta-glucosidase | GBA3 | 20 | 4IID, 4IIC, 4IIF, 1W3J, 2J7B, 2J7D, 2J75, 2J79, 2WC3, 2CBU, 2WC4, 1OIM, 2J7E, 2J7F, 2J7G, 1UZ1, 2CES, 2J78, 2CET, 2CBV |
| Biotin carboxylase | BC | 10 | 3V7S, 2W6O, 2W6M, 2W6Q, 2V5A, 3JZI, 2W71, 2V59, 2W6Z, 3EFR |
| Protein kinase A | PKA | 45 | 2OH0, 3L9M, 2GNH, 2JDT, 3L9N, 3DND, 2GNI, 2C1B, 2GU8, 1YDS, 4C35, 2JDV, 3ZO4, 2UW4, 1XH6, 2OJF, 2UW5, 2VO7, 3OW3, 2GNJ, 4C36, 2JDS, 1SVH, 3ZO2, 2UW8, 2UW7, 3DNE, 1Q8W, 1SVE, 2GNF, 3AMB, 1XH4, 3AMA, 2UW6, 2C1A, 3L9L, 3VQH, 3ZO1, 2ERZ, 2UW0, 1VEB, 1Q8T, 1YDT, 1Q8U, 1YDR |
| Dual-specificity phosphatase | DSP | 33 | 4AN9, 4C4E, 3WIG, 4C4I, 3ZLW, 4AN2, 4C4J, 3V04, 4ARK, 4C4H, 4ANB, 3H9F, 4AN3, 3ZLS, 2ZMD, 2WU6, 3W1F, 4BHZ, 3ZLY, 4BI1, 3PP1, 3GFW, 3KVW, 3V01, 4BI0, 4C4F, 4C4G, 4LMN, 3OS3, 3VQU, 3ZM4, 3ANQ, 3ANR |
