# Supplementary figures and images for: GraphscoreDTA: optimized graph neural network for protein–ligand binding affinity prediction
Source: Bioinformatics. 2023 May 24;39(6):btad340. doi: 10.1093/bioinformatics/btad340 (PMC10243863; doi:10.1093/bioinformatics/btad340)

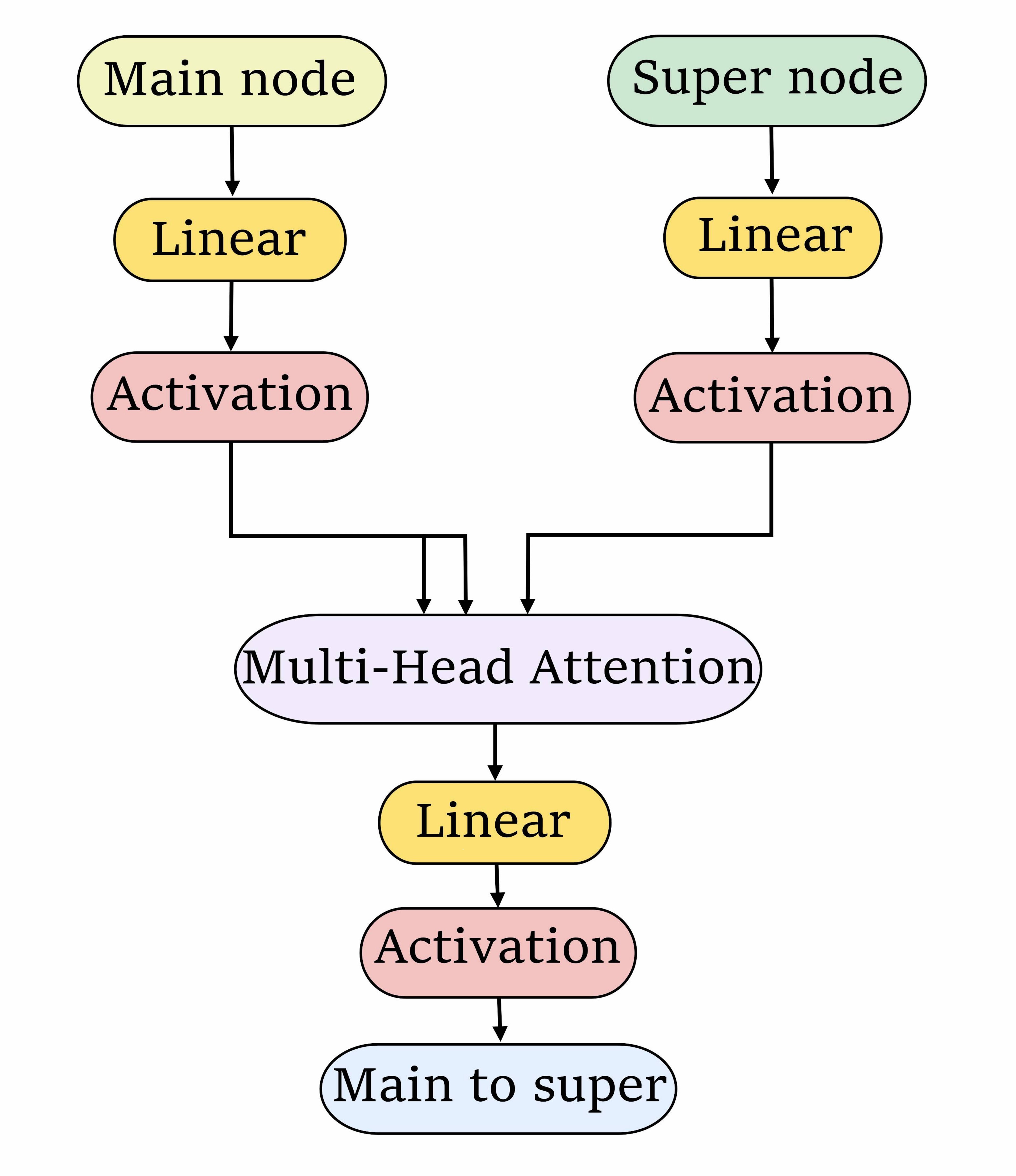

Supplement: btad340_Supplementary_Data [file btad340_supplementary_data.zip › FigS2_DPI350.jpg]

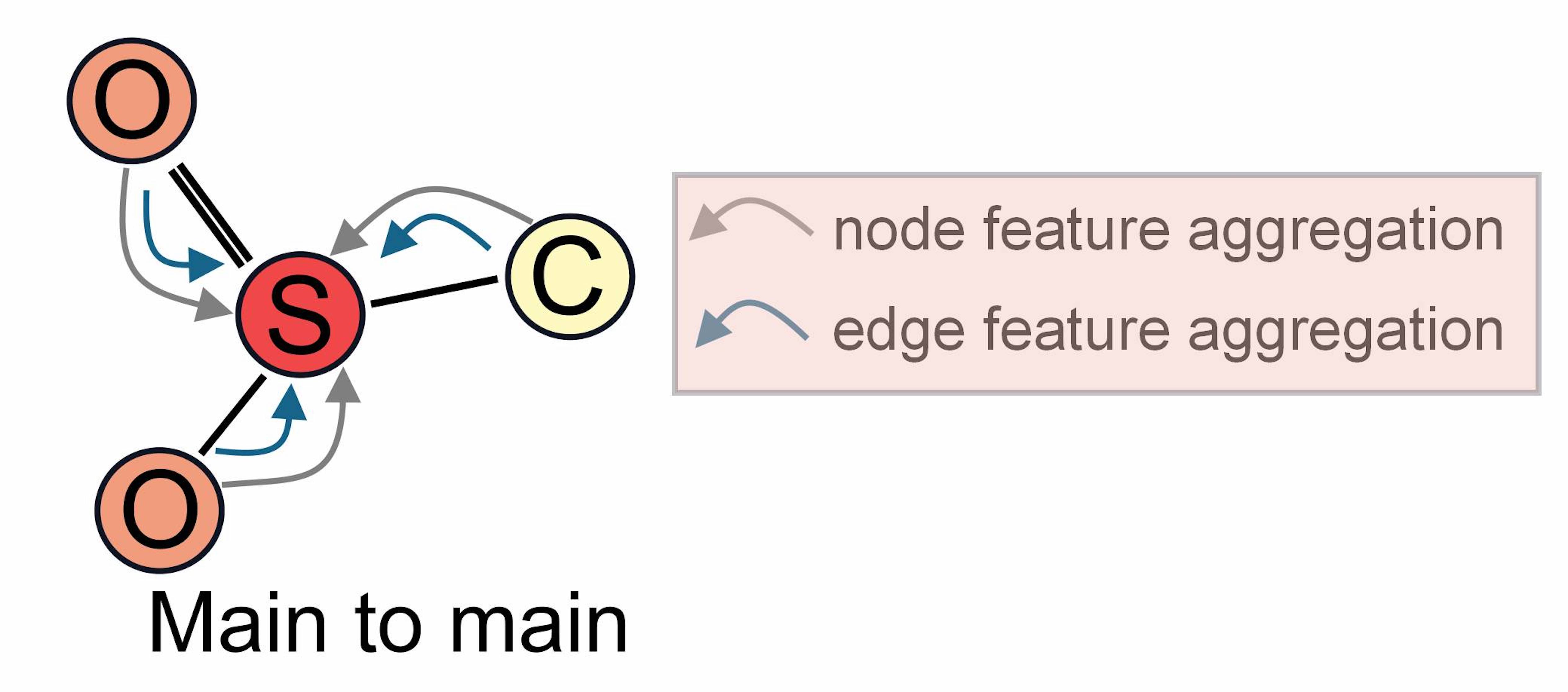

Supplement: btad340_Supplementary_Data [file btad340_supplementary_data.zip › FigS3_DPI350.jpg]

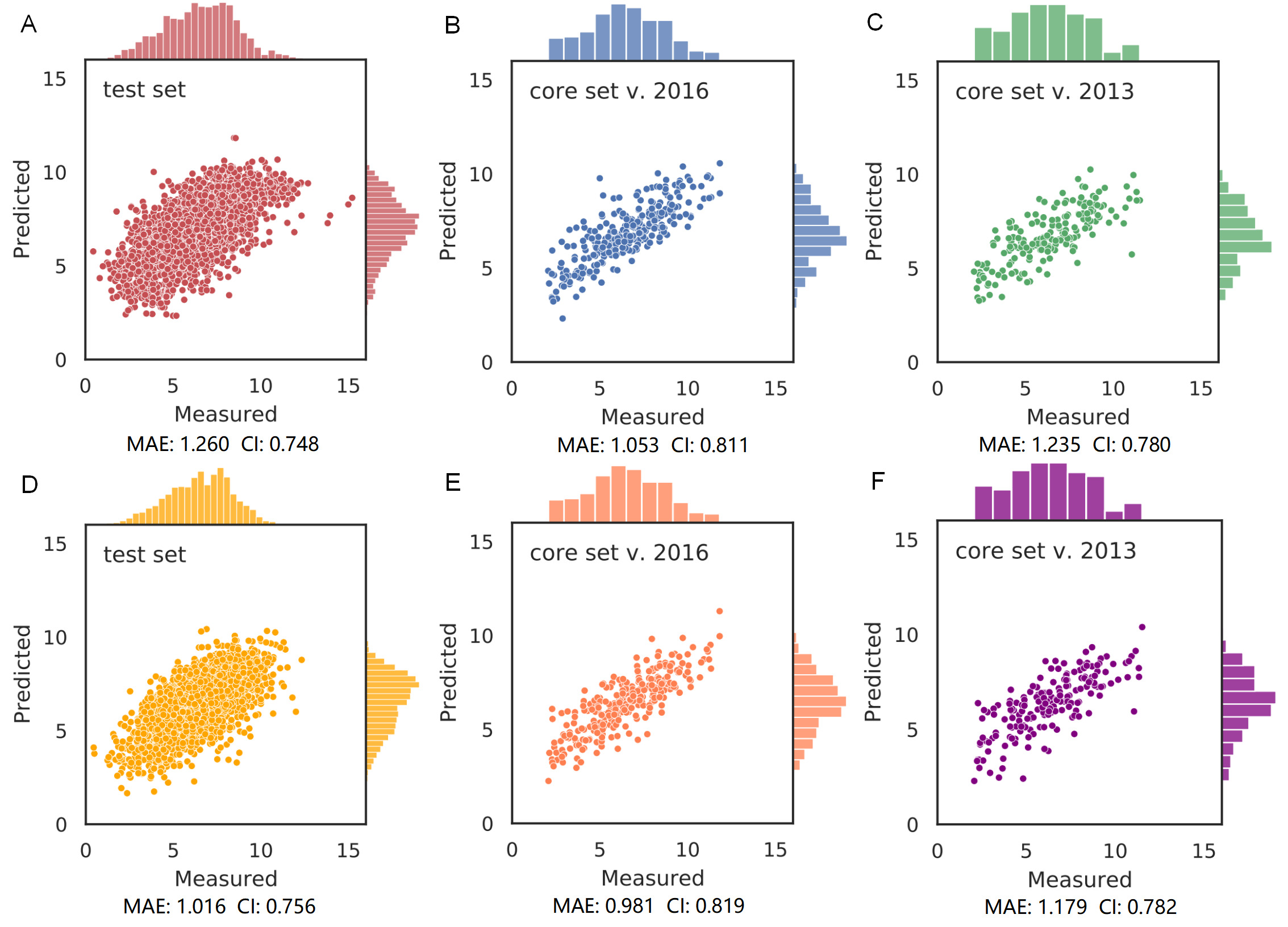

Supplement: btad340_Supplementary_Data [file btad340_supplementary_data.zip › FIGS4_DPI350.jpg]

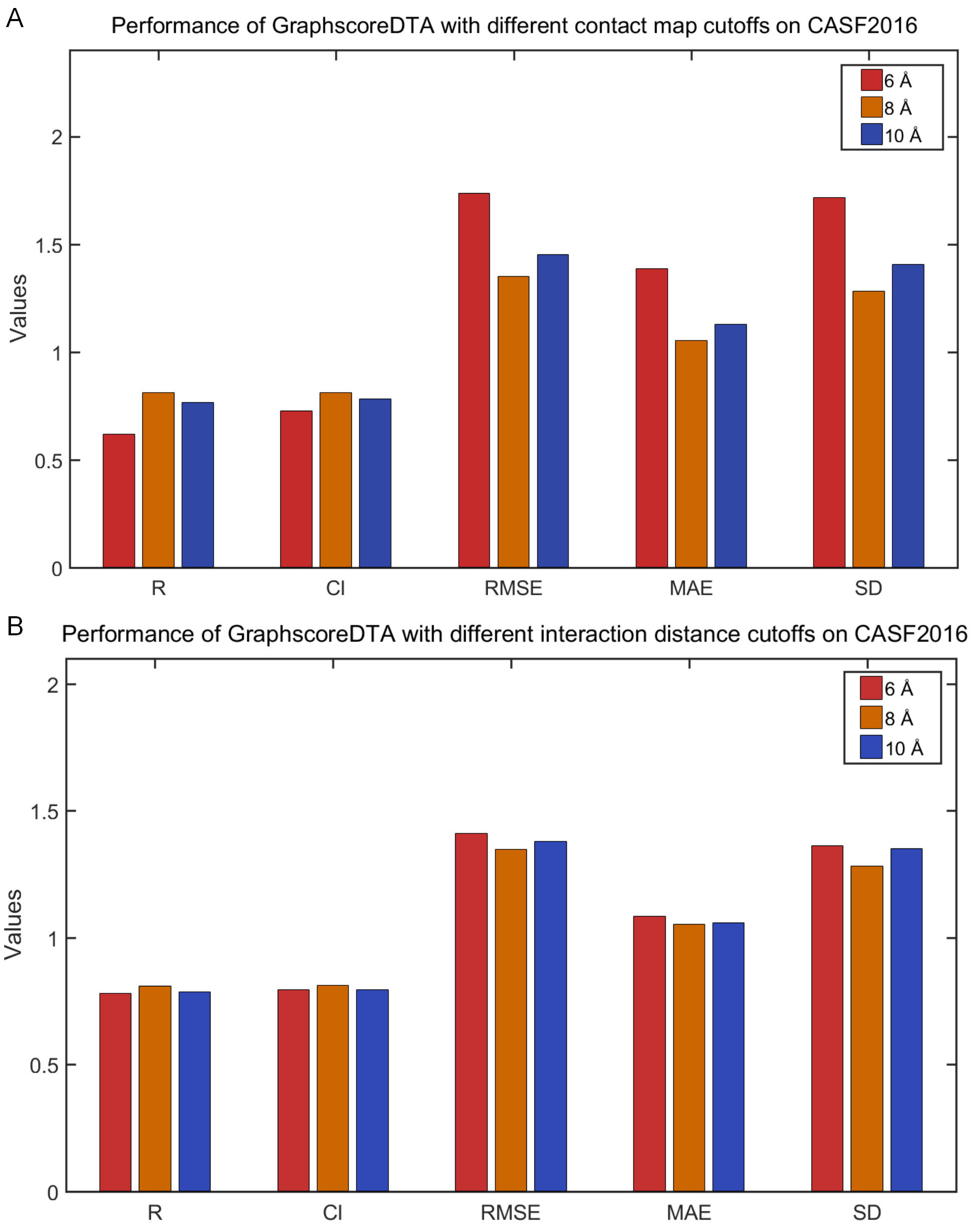

Supplement: btad340_Supplementary_Data [file btad340_supplementary_data.zip › figS5_DPI350.jpg]

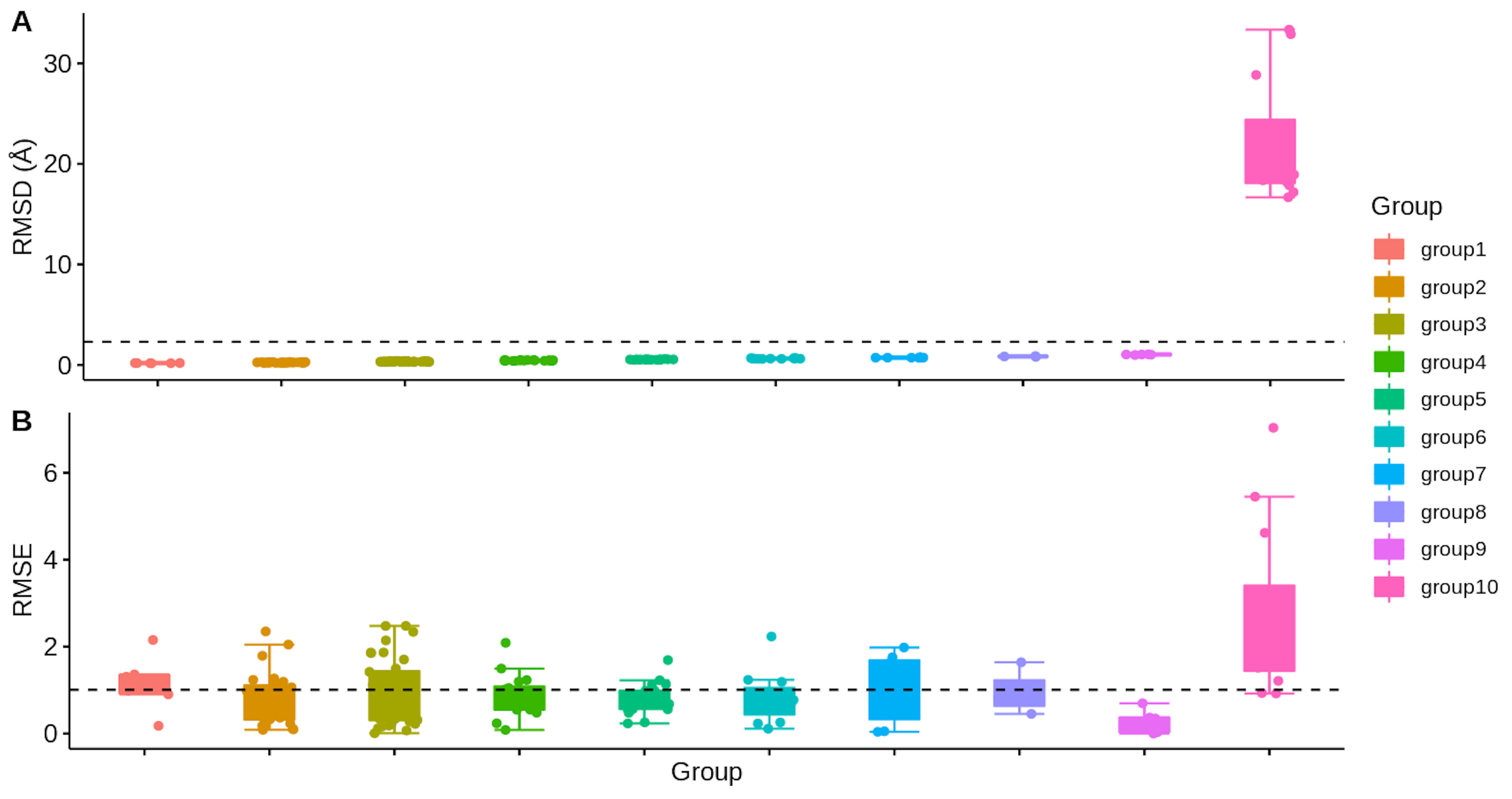

Supplement: btad340_Supplementary_Data [file btad340_supplementary_data.zip › FigS6_DPI350.jpg]

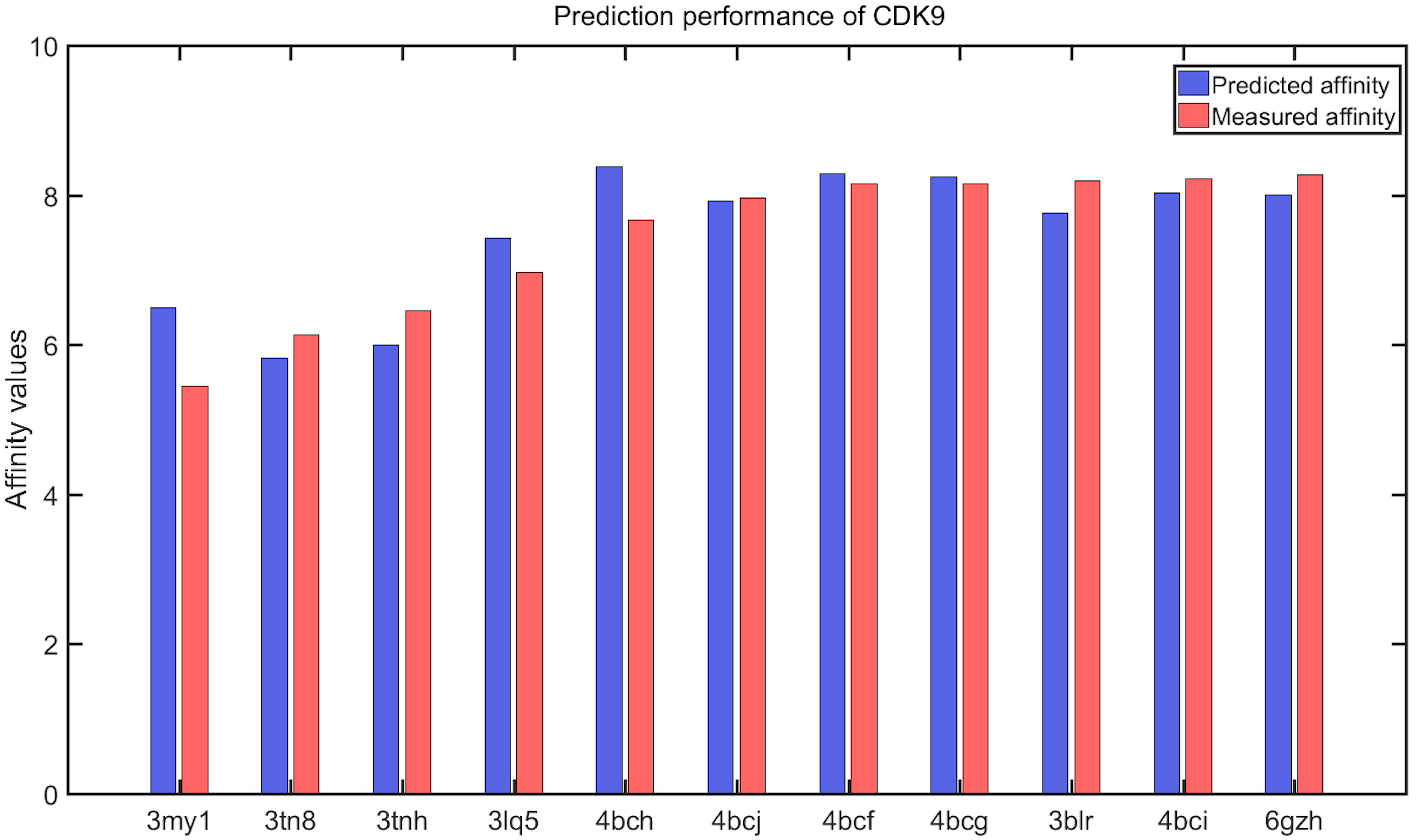

Supplement: btad340_Supplementary_Data [file btad340_supplementary_data.zip › FigS7_DPI350.jpg]

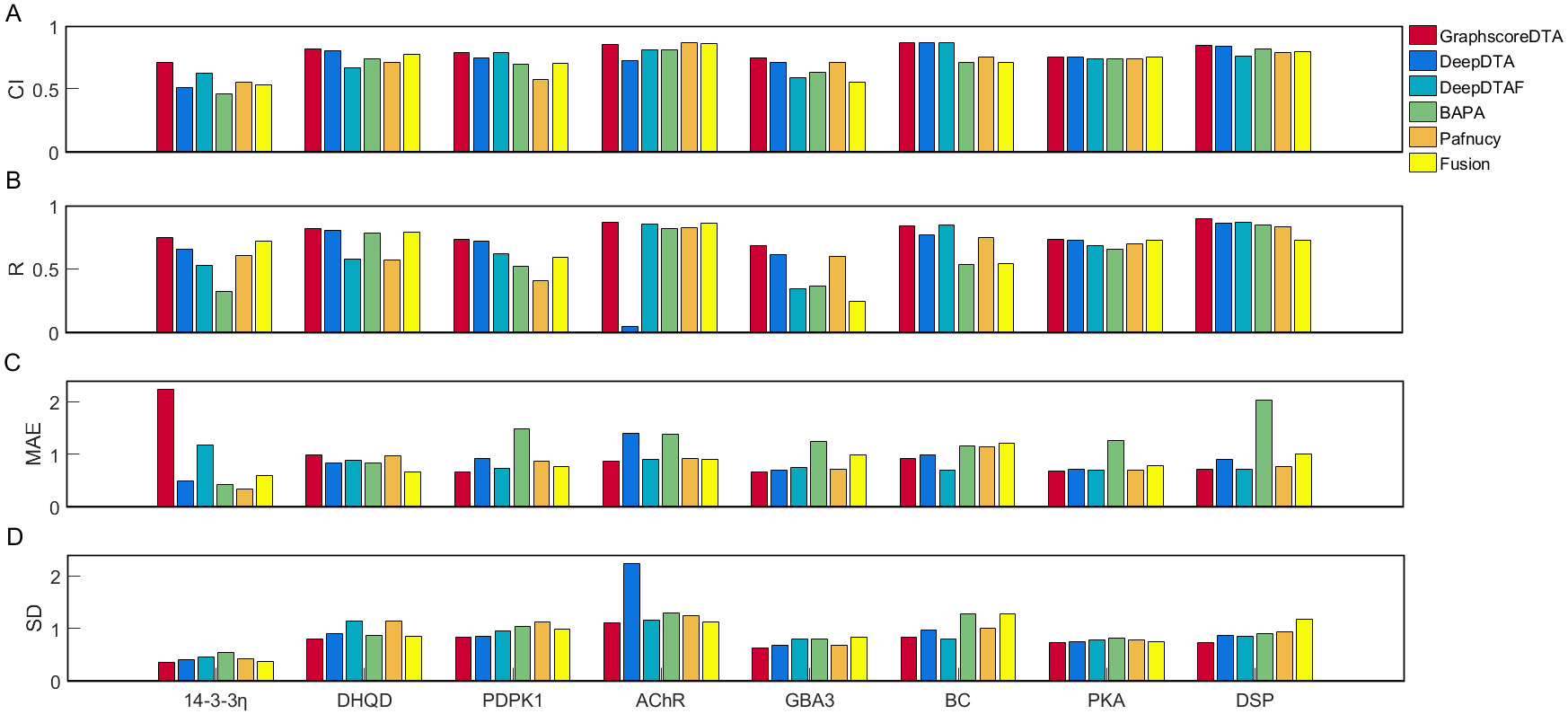

Supplement: btad340_Supplementary_Data [file btad340_supplementary_data.zip › FigS8_DPI350.jpg]

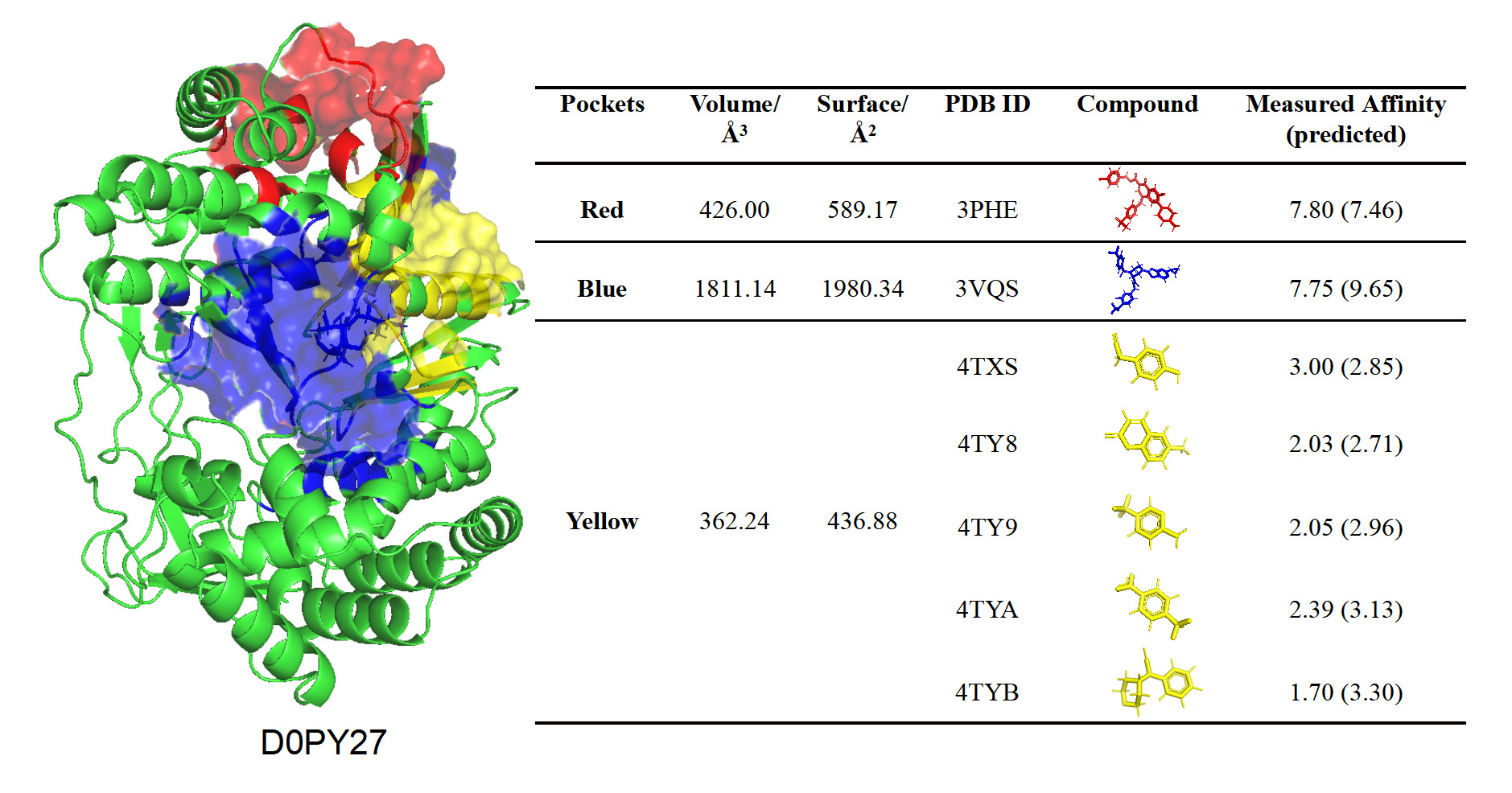

Supplement: btad340_Supplementary_Data [file btad340_supplementary_data.zip › figS9_DPI350.jpg]

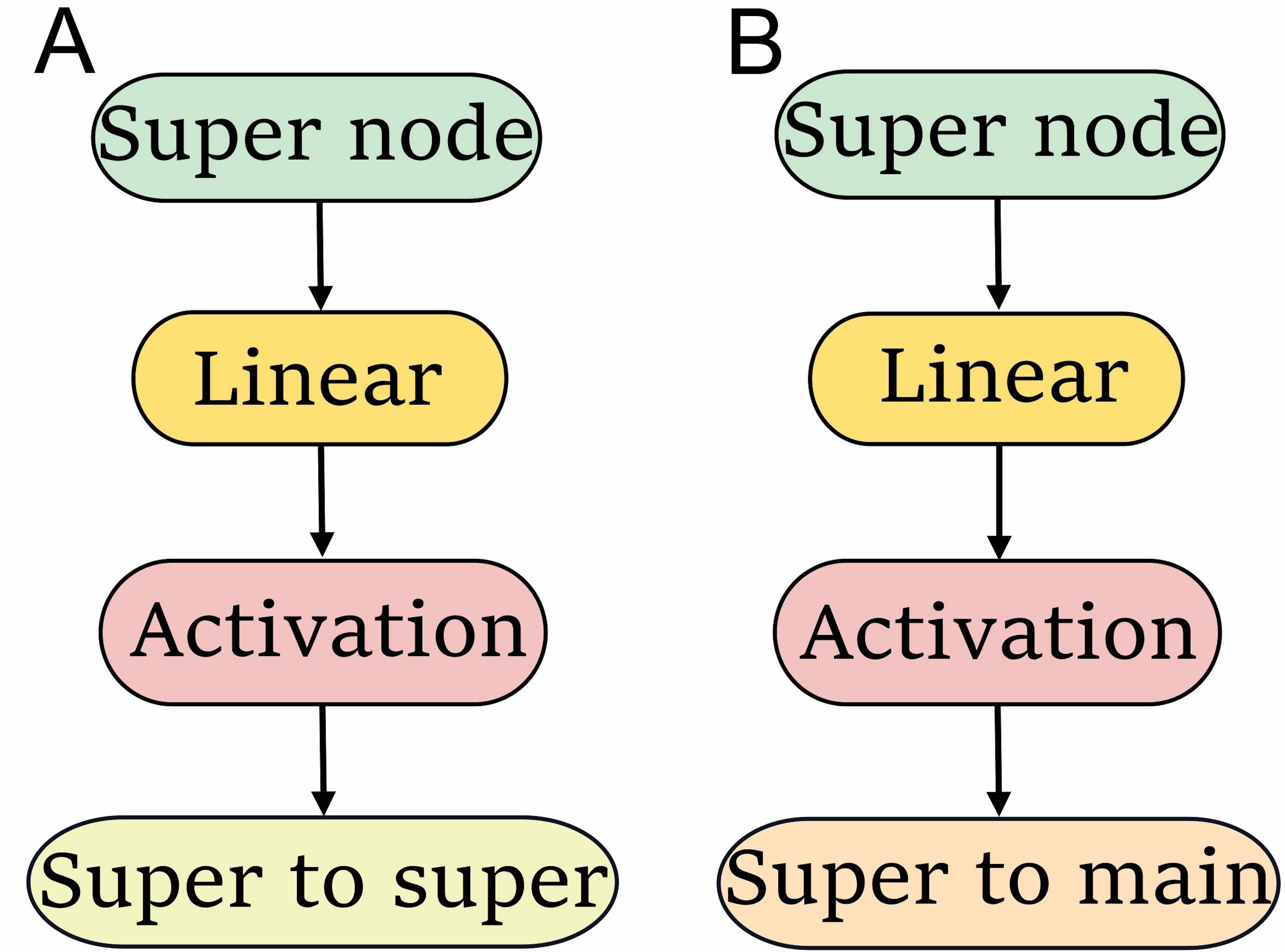

Supplement: btad340_Supplementary_Data [file btad340_supplementary_data.zip › FigS1_DPI350.jpg]
